# Oxidized and Unsaturated: Key Organic Aerosol Traits Associated with Cellular Reactive Oxygen Species Production in the Southeastern United States

**DOI:** 10.1021/acs.est.3c03641

**Published:** 2023-09-12

**Authors:** Fobang Liu, Taekyu Joo, Jenna C. Ditto, Maria G. Saavedra, Masayuki Takeuchi, Alexandra J. Boris, Yuhan Yang, Rodney J. Weber, Ann M. Dillner, Drew R. Gentner, Nga L. Ng

**Affiliations:** † Department of Environmental Science and Engineering, School of Energy and Power Engineering, Xi’an Jiaotong University, Xi’an, Shaanxi 710049, China; ‡ School of Chemical and Biomolecular Engineering, Georgia Institute of Technology, Atlanta, Georgia 30332, United States; § School of Earth and Atmospheric Sciences, Georgia Institute of Technology, Atlanta, Georgia 30332, United States; ∥ Department of Chemical and Environmental Engineering, Yale University, New Haven, Connecticut 06511, United States; ⊥ Air Quality Research Center, University of California Davis, Davis, California 95618, United States; # School of Civil and Environmental Engineering, Georgia Institute of Technology, Atlanta, Georgia 30332, United States

**Keywords:** organic aerosol, secondary organic aerosol, oxygenated organic aerosol, reactive oxygen species, toxicity, aerosol health effects, aerosol mass spectrometer

## Abstract

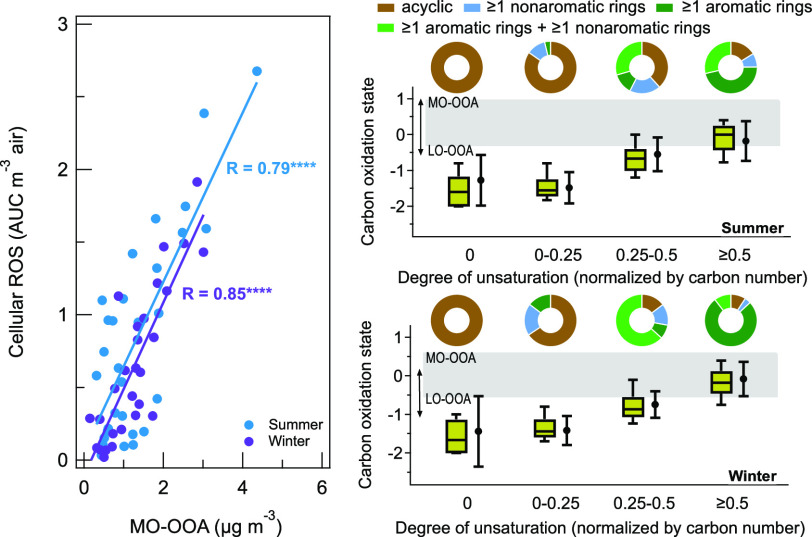

Exposure to ambient fine particulate matter (PM_2.5_) is associated with millions of premature deaths annually. Oxidative stress through overproduction of reactive oxygen species (ROS) is a possible mechanism for PM_2.5_-induced health effects. Organic aerosol (OA) is a dominant component of PM_2.5_ worldwide, yet its role in PM_2.5_ toxicity is poorly understood due to its chemical complexity. Here, through integrated cellular ROS measurements and detailed multi-instrument chemical characterization of PM in urban southeastern United States, we show that oxygenated OA (OOA), especially more-oxidized OOA, is the main OA type associated with cellular ROS production. We further reveal that highly unsaturated species containing carbon–oxygen double bonds and aromatic rings in OOA are major contributors to cellular ROS production. These results highlight the key chemical features of ambient OA driving its toxicity. As more-oxidized OOA is ubiquitous and abundant in the atmosphere, this emphasizes the need to understand its sources and chemical processing when formulating effective strategies to mitigate PM_2.5_ health impacts.

## Abstract 2

We find that oxygenated organic aerosol (OA) is the largest contributor to OA toxicity, in which highly unsaturated species containing carbon−oxygen double bonds and aromatic rings are the key chemical features driving its toxicity.

## 1 Introduction

Ambient particulate matter (PM; also referred to as atmospheric aerosol) is a chemically complex mixture of particles that are generated via both natural and anthropogenic activities. Exposure to fine PM with a diameter smaller than 2.5 μm (PM_2.5_) has been associated with adverse health effects and up to nine million deaths per year.^1−3^ Yet, due to the complexity of PM_2.5_, it remains unclear which components or characteristics of PM_2.5_ best determine its overall toxicity. There is an increasing recognition that the mass-based regulatory metric (PM_2.5_ mass concentration) may not be sufficient to evaluate its health impacts. Investigation of the chemical components and biological mechanisms driving PM_2.5_ toxicity is critically needed.^4−6^

Increased reactive oxygen species (ROS) production and the resulting oxidative stress is a possible mechanism for PM_2.5_ toxicity, as suggested by multiple studies.^7−10^ Accordingly, PM_2.5_-induced ROS production has been an increasingly used metric to evaluate PM_2.5_ toxicity.^11−13^ Cell-free, chemical-based assays (e.g., dithiothreitol assay) are often used to assess the chemical oxidative potential of PM_2.5_.^4,14^ These assays are less time- and resource-intensive than cellular assays, but they cannot capture ROS generated by cells upon PM_2.5_ stimulation, which could account for a significant fraction of cellular ROS.^15,16^ In contrast, cellular assays allow for capturing both chemically and biologically generated ROS, as well as elucidating the mechanisms of cellular signaling, response, and damage. Hence, measurement of cellular ROS production offers a more comprehensive evaluation of the ability of PM_2.5_ to induce oxidative stress and subsequent negative biological effects.

Some PM_2.5_ components such as organic compounds and transition metals are suggested to be important in cellular ROS production.^13,17^ Transition metals in PM_2.5_ can be quantified by well-established analytical techniques, such as inductively coupled plasma-mass spectrometry (ICP-MS), though their various chemical and physical forms may further affect their toxicity. In contrast, organic aerosol (OA), which constitutes a substantial fraction (20–90%) of fine PM, contains hundreds of thousands of individual organic compounds.^18−20^ This complexity makes it extremely challenging to identify the OA components that are responsible for cellular ROS production.

Alternatively, aerosol mass spectrometry (AMS) with factor analysis is a widely used approach to differentiate major types of OA by sources and properties, based on their unique mass spectral signatures.^18,21^ Common OA types that have been observed around the world include hydrocarbon-like OA (HOA, a surrogate of primary OA from fossil fuel combustion); oxygenated OA (OOA, a surrogate of secondary OA (SOA) that is formed by atmospheric oxidation of natural and anthropogenic emissions, often can be further separated into more-oxidized OOA (MO-OOA) and less-oxidized OOA (LO-OOA)); biomass burning OA (BBOA); and cooking OA (COA).^4,18,22,23^ Among these, OOA is ubiquitous and is a dominant fraction of OA in the atmosphere.^18,22,24^ Other analytical techniques, such as Fourier transform infrared (FT-IR) spectrometry, liquid chromatography with tandem mass spectrometry (LC–MS/MS), and chemical ionization mass spectrometry coupled with a filter inlet for gases and aerosols (FIGAERO-CIMS), are complementary approaches to AMS that provide considerable insights into OA structural features and functional groups.^25,26^ While a combination of these analytical techniques allows for highly detailed chemical characterization of OA, these tools have not been employed alongside toxicological studies to investigate the ability of ambient OA to induce cellular ROS production and to identify the key toxic components and chemical features of OA.

A few laboratory studies have investigated the cellular toxicity of SOA formed from a single gaseous precursor under well-controlled conditions.^27−32^ These studies show that the oxidative properties of SOA largely depend on SOA precursors and reaction conditions. SOA formed from aromatic-ring-containing precursors is found to induce higher levels of cellular ROS production and inflammatory response.^28,30^ Laboratory investigations also suggest that atmospheric aging processes can enhance SOA cellular toxicity.^27,28,31,32^ Consistent with these cellular studies, increasing aerosol toxicity with atmospheric aging has also been noted utilizing acellular assays, such as dithiothreitol assay.^33,34^ Field investigations have found that some species such as quinone-type compounds and organic peroxides in OA significantly contribute to cellular ROS production.^13,35−38^ However, detailed chemical characterization of ambient PM is still needed to determine the types and chemical features of OA that drive cellular ROS production.

In this study, to elucidate the linkage between PM_2.5_ constituents, chemical functionality, and cellular ROS production upon exposure to PM_2.5_, we measure cellular ROS with an alveolar macrophage assay for comparison to detailed PM_2.5_ chemical speciation across multiple instruments. The field observations are performed at an urban site in Atlanta (Jefferson Street, SEARCH network^39,40^) across two seasons with influences from both biogenic and anthropogenic emissions. We resolve the major types of OA using factor analysis of AMS data and determine the functional groups and chemical composition of OA at the molecular level. Through these integrated cellular ROS measurements and chemical characterization, we show that OOA, especially more-oxidized OOA (MO-OOA), is the main contributor to cellular ROS production upon PM_2.5_ exposure. We further provide evidence that the highly unsaturated species containing carbon–oxygen double bonds and aromatic rings in OOA could explain the predominant contribution of OOA to cellular ROS production.

## 2 Methods

The measurements took place from July 19 to August 25, 2017 (summer) and January 15 to February 11, 2018 (winter) at Jefferson Street, which is situated in urban Atlanta and is surrounded by a mixed residential and commercial neighborhood. The sampling site is part of the Southeastern Aerosol Research and Characterization (SEARCH) network and has been extensively used in characterizing urban Atlanta air quality, measuring physical and chemical properties of PM, and linking oxidative properties of PM to epidemiological studies.^39,41−44^

### 2.1 PM Chemical Characterization

#### 2.1.1 Online Measurements

A high-resolution time-of-flight aerosol mass spectrometer (HR-ToF-AMS, Aerodyne Research Inc.) was used to characterize the chemical composition of submicron nonrefractory PM (NR-PM_1_). Positive Matrix Factorization (PMF) analysis was performed on the high-resolution organic mass spectra (*m*/*z* 12–150) using PMF Evaluation Toolkit (PET v.2.0x) to determine the sources of OA. Results from the winter data have been reported in Joo et al.^45^ In addition, a filter inlet for gases and aerosols coupled to an iodide-adduct chemical ionization mass spectrometer (FIGAERO-CIMS) was deployed to measure the gas-phase and particle-phase oxidized organic compounds.^26,46,47^ The details of instrument operation and data processing are provided in the Supporting Information.

#### 2.1.2 Offline Measurements

Multiple sets of filter samples (Table S1) were collected during summer and winter for chemical and cellular analysis. PM_2.5_ samples (24 h) were collected onto prebaked quartz filters (Pallflex Tissuquartz) using a high-volume sampler (HiVol, Thermo Anderson, flow rate 1.13 m^3^ min^–1^). PM_2.5_ mass concentration was monitored continuously by a tapered element oscillating microbalance (TEOM, Thermo Scientific TEOM 1400a). The average PM_2.5_ mass concentration was 12 ± 4 μg/m^3^ in summer and 9 ± 3 μg/m^3^ in winter during the sampling periods. The collected filter samples were wrapped in prebaked aluminum foil and stored at −20 °C until extraction and analysis. Water-soluble transition metals (Fe, Cu, Mn, Cr, and Zn) of PM_2.5_ samples were measured using ICP-MS (Agilent 7500a series, Agilent Technologies, Inc.).^48^ Prior to analysis, a punch (5.1 cm^2^) of filters was sonicated in deionized water for 30 min. After sonication, the extract was filtered with a 0.45 μm PTFE syringe filter (Fisherbrand) and acid-preserved by adding concentrated nitric acid (70% to a final concentration of 2% (v/v)).

A set of daytime and nighttime PM_10_ samples were collected during the summer and winter sampling periods for LC–MS/MS analysis. In summer, daytime samples were collected from 9:00 to 19:00 and in winter from 9:00 to 17:00. In summer, nighttime samples were collected from 22:30 to 5:00 and in winter from 21:00 to 5:00. Samples were collected at 16.67 L/min on 47 mm Teflon filters (Tisch Environmental). Filters were stored in sealed Petri dishes at −30 °C until extraction and analysis. For LC–MS/MS analysis, filters were first extracted in 2 mL of methanol (>99.9%, Sigma-Aldrich), with 60 min of sonication at room temperature. Extracts were then concentrated to 200 μL under gentle nitrogen flow. Extracts were analyzed via liquid chromatography (Agilent 1260 Infinity) with both positive and negative mode electrospray ionization and quadrupole time-of-flight mass spectrometry (Agilent 6550 Q-TOF). The detailed method descriptions of LC–MS/MS analysis are provided in the Supporting Information. The daytime and nighttime profiles of organic molecular features and functional groups determined by LC–MS/MS have been reported in previous studies.^25,49^

PM_1_ samples were collected on 47 mm PTFE filters with 2 μm pore size (Measurement Technology Laboratories) downstream of a PM_1_ cyclone during the winter for organic functional group quantification by FT-IR spectrometry. A mass flow control valve was set to 16.7 L/min. Samples were collected for 24 h on some days and for 8 h daytime and 8 h nighttime samples on other days. Filters were stored cold to minimize loss of volatile species. The filters were directly analyzed using a Bruker Tensor II FT-IR spectrometer (Bruker Optics, Inc.), operated in transmission mode, with a liquid-nitrogen-cooled mercury cadmium telluride detector. The detailed method descriptions of FT-IR spectrometry analysis are provided in the Supporting Information. The organic functional group profiles determined by FT-IR spectrometry have been reported in Boris et al.^50^

### 2.2 Intracellular ROS Measurements

Murine alveolar macrophages (MH-S, ATCC, CRL-2019) were maintained in RPMI-1640 media (ATCC) supplemented with 10% feta bovine serum (FBS, VWR), 1% penicillin–streptomycin (VWR), and 50 μM β-mercaptoethanol (BME, Sigma-Aldrich) at 37 °C and 5% CO_2_. Intracellular ROS levels were measured following the methodology established in our previous study.^11^ Briefly, the assay consists of five steps: (1) pretreatment of 96-well plates with 10% FBS in phosphate-buffered saline (PBS), (2) seeding of cells at 2 × 10^4^ cells per well, (3) incubation with a ROS probe (carboxy-H_2_DCFDA, Molecular Probes C-400), (4) exposure of cells to PM samples for 24 h, and (5) detection of ROS using a microplate reader (BioTek Synergy H4, excitation/emission 485/525 nm). Control (RPMI-1640 media supplemented with FBS) and positive control (100 μM H_2_O_2_) samples were conducted for each cellular ROS measurement. Alveolar macrophages are the first line of defense against inhaled environmental contaminants. This cell line has been used for measuring cellular ROS production upon PM exposure in a number of prior studies.^11,15,30−32,36,51^

PM_2.5_ filters were extracted following the established protocol where the filters were submerged in RPMI-1640 media and sonicated for 30 min.^11^ Note that some studies suggest that antioxidants and ligands in the lung fluid may consume or complex with PM components.^52,53^ When these effects of the lung fluid are not considered, the potency of PM-induced oxidative stress may be overestimated. However, these studies are based on acellular assays. Future work is warranted to explore the effects of antioxidants and ligands in the lung fluid on cellular ROS measurements. Extracts were filtered with 0.45 μm PTFE syringe filters (Fisherbrand) and supplemented with FBS prior to cell exposure. For each PM_2.5_ filter sample, ROS production was measured over 10 dilutions in triplicate and expressed as a fold increased over control cells (i.e., ROS produced from probe-treated control cells). A representative dose–response curve is shown in Figure S1. The area under the dose–response curve (AUC) is used to represent ROS production, as the AUC has been shown to be the most robust metric for evaluating ROS production of PM samples.^11^ The AUC was calculated over the same dose range (2 μg) for all PM_2.5_ samples. This dose range was used based on estimating the dose delivered to the alveolar region for 24 h human exposure in Atlanta.^54^ Note that the short-lived reactive and labile species in PM_2.5_ samples could be lost during filter collection, storage, and extraction. In addition, water-soluble PM extracts contributing to cellular ROS production were investigated while unextracted PM components may also trigger cellular ROS production and warrant further studies.

### 2.3 Multiple Linear Regression Model

A multiple linear regression model (MLRM) was applied to identify the main PM components that contributed to cellular ROS production. AMS-identified OA types and five water-soluble transition metals (Fe, Cu, Mn, Cr, and Zn) were selected as the independent variables, since these species are widely suggested to play a role in cellular ROS production.^10,11,13,17,55^ Inorganic ions were excluded as they are components of RPMI-1640 media and previous studies have indicated that they do not induce cellular ROS production.^4,30^ A forward selection regression was used, and the Akaike information criterion (AIC) and the Bayesian information criterion (BIC) were calculated to determine the variables to be included in the model. In general, the AIC was used as the decisive metric and the predictors with the lowest AIC were selected. If adding an extra predictor leads to a comparable AIC (difference within 2%) but a higher BIC, the extra predictor will not be included in the model. To assess the uncertainty and sensitivity of the MLRM to the input data, we combined different randomly selected OA types and metal data and performed bootstrap analysis (1000 runs). The same method was applied to resolve the contribution of five functional groups (results from FT-IR analysis) to OOA, AMS-identified OA types, and total AMS OA.

The resolved predictors were further diagnosed for collinearity by examining tolerance and the variance inflation factor (VIF) of MLRM-resolved predictors. A tolerance lower than 0.1 and a VIF higher than 10 were used to detect the occurrence of collinearity between two independent variables, as shown in Table S2. No collinearity was found for the MLRM-resolved predictors for cellular ROS. In the MLRM for OOA and LO-OOA, a collinearity between non-acid and non-oxalate carbonyls (naCO) and alcohols (aCOH) was found. We further compared the results from either excluding naCO or aCOH. We found a greater coefficient of determination (*R*^2^) between modeled and measured results when excluding aCOH than naCO. Thus, to avoid a bias from collinearity, aCOH was not included in the final MLRM-resolved predictors for OOA and LO-OOA.

### 2.4 Statistical Analysis

Linear regressions between cellular ROS and PM components were evaluated using Pearson’s correlation coefficients (Pearson R). Hierarchical cluster analysis based on the Pearson R matrix was performed using a custom MATLAB script. Significance of differences for carbon oxidation states between compounds in each DU/C (degree of unsaturation normalized by carbon number) compound group was evaluated using the one-way ANOVA test (Table S3). The calculation methods of DU/C and carbon oxidation states from LC–MS/MS data, FIGAERO-CIMS data, and AMS-identified OA types are provided in the Supporting Information. Significance was determined using two-tailed tests of *p* values with a confidence interval of 95%. The statistical analysis was performed using GraphPad Prism 9.

## 3 Results and Discussion

### 3.1 Exploring Relationships between Cellular ROS Production, OA Types, Transition Metals, and PM_2.5_ Mass Concentration

Similar to prior field studies conducted around the world, we observe that OA is the most abundant component in PM_2.5_ and OOA is the dominant type of OA in Atlanta (Figure S2).^6,18,56^ To yield insights into the sources and chemical properties of OA that are associated with PM_2.5_ toxicity, we investigated the relationship between OA types (determined via factor analysis of AMS data) and cellular ROS production. To determine whether OA or transition metals are more closely correlated to cellular ROS production, five water-soluble transition metals (Fe, Cu, Mn, Cr, and Zn) were included in the analysis because of their high redox activity and/or high abundance.^11,13,14,57^ Finally, PM_2.5_ mass concentration was also included as it is the current regulatory metric for PM_2.5_ health impacts.

**Figure 1:**
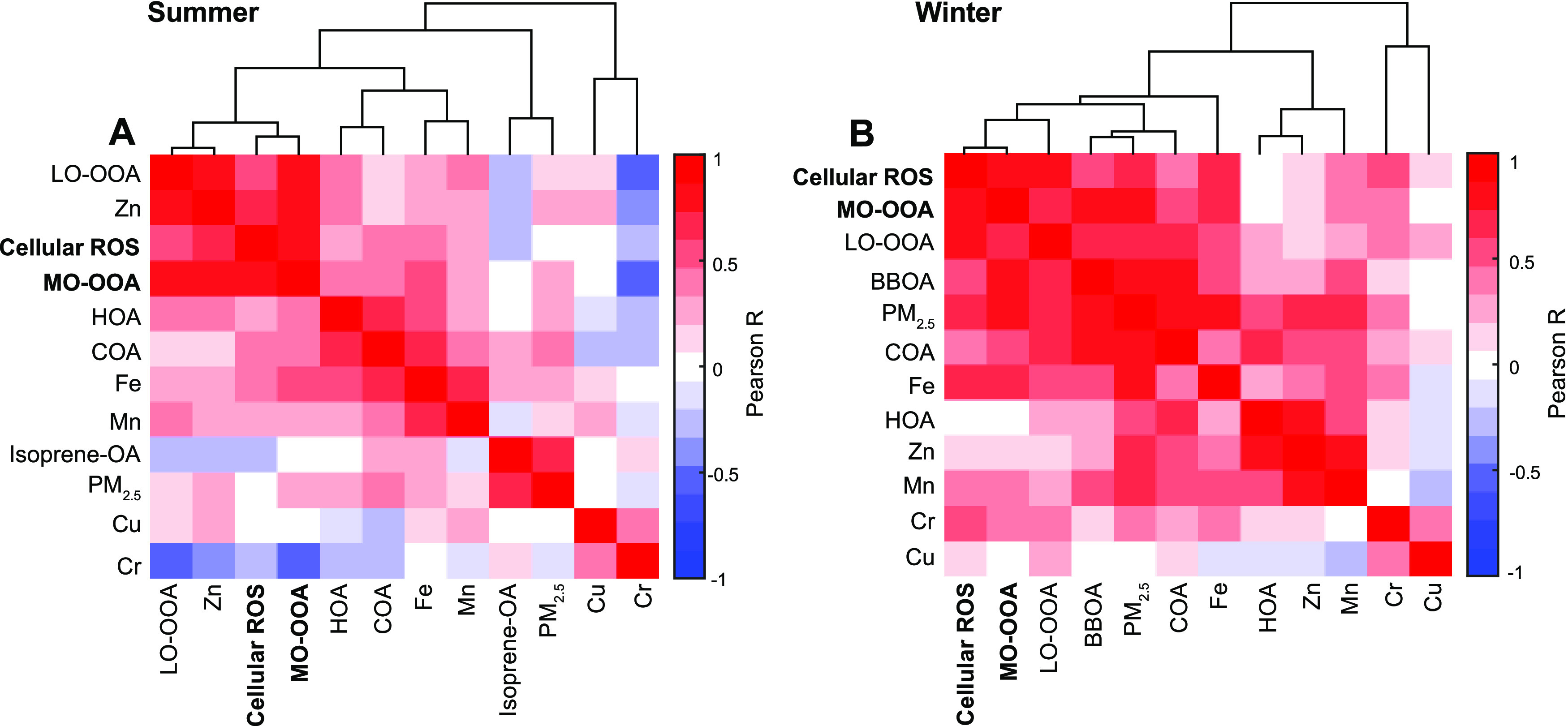
Clustergrams of cellular ROS and selected PM components. Summer (A) and winter (B) hierarchical clustering performed using the Pearson correlation coefficient (Pearson R) between key variables. The sample size for summer and winter is 29 and 28, respectively. The input data include cellular ROS (AUC m^–3^ air), mass concentrations (μg m^–3^) of PM_2.5_ and selected PM components. The selected PM components include AMS-identified OA types (MO-OOA, LO-OOA, HOA, COA, isoprene-OA, and BBOA) and five water-soluble transition metals (Fe, Cu, Mn, Cr, and Zn). A version of this figure with R values shown on each square is provided in Figure S8. A version merging the data from both seasons is provided in Figure S9 (“0” is used for the unidentified OA type in each season).

Figure 1 shows the hierarchical cluster analysis results for cellular ROS (AUC, AUC m^–3^ air; see Section 2) with OA types, transition metals, and PM_2.5_ mass concentration, according to their Pearson *R* matrix. In both seasons, cellular ROS is first clustered with MO-OOA, meaning they are highly correlated and exhibit lower Pearson *R* values with other variables. The cluster is then merged with LO-OOA (winter, Figure 1B) or the group containing LO-OOA (summer, Figure 1A). These results indicate a strong association between cellular ROS and OOA (often subdivided into MO-OOA and LO-OOA according to their degree of atmospheric oxidation), especially for MO-OOA (*R*_summer_ = 0.77 and *R*_winter_ = 0.85, *p* < 0.0001, scatterplots are provided in Figure S3). It is notable that the association between cellular ROS and PM_2.5_ mass concentration does not stand out compared to specific PM components such as MO-OOA and LO-OOA, indicating cellular ROS production is not simply driven by PM_2.5_ mass concentration.

LO-OOA is fresh SOA largely formed from oxidation of organic gases.^58^ MO-OOA is highly oxidized and can be formed from multiple sources and pathways, including photochemical aging of fresh SOA, aqueous processes, chemical processing during long-range transport, partitioning of highly oxygenated molecules (HOMs), and entrainment of aged SOA from the residual layer.^18,59−62^ Although the strong association between cellular ROS and MO-OOA does not necessarily establish causation, previous laboratory work has reported that aged SOA induces higher cellular ROS production compared to fresh SOA,^27,31,32^ whereas only SOA formed from a single gaseous precursor was investigated in these laboratory studies. The impact of atmospheric aging processes on the cellular ROS production of ambient PM_2.5_ has not been well explored. The strong association between cellular ROS and MO-OOA reveals that the cellular ROS production of ambient PM_2.5_ is highly influenced by its degree of oxidation and aging processes. It is noted that cellular ROS production of summer and winter samples is consistently clustered primarily with OOA with less influence by soluble forms of transition metals, suggesting that the ROS production is driven more by OOA than transition metals. Yet, the capability of metals in complexing with organic compounds to generate ROS and enhance aerosol aging should be noted.^32,63−65^ Also, ROS production may depend on the redox coupling of metals and organics, which could be site-specific.^10,66^

### 3.2 Establishing Oxygenated OA as the Main Contributor to Cellular ROS Production

To further examine and quantify the contributions of OA types and transition metals to cellular ROS production, we employed an MLRM. The AMS-identified OA types (MO-OOA, LO-OOA, HOA, COA, and isoprene-OA (summer) or BBOA (winter)) and five water-soluble transition metals (Fe, Cu, Mn, Cr, and Zn) were used as independent variables. The measured cellular ROS is well captured by the model with overall *R*^2^ between modeled and measured results greater than 0.7 in individual seasons (Table 1). The MLRM regression coefficients (unstandardized coefficients) in Table 1 are the observed cellular response per mass of the specific species. Higher MLRM regression coefficients are found for transition metals than OA, which is consistent with prior work.^4,55^

**Table 1:** MLRM-Resolved Regression Coefficients and Standard Deviations of Each Independent Variable for Cellular ROS^a^

		sample size	MO-OOA	LO-OOA	isoprene-OA	HOA	COA	Fe	Cu	Mn	intercept	*R*^2^
cellular ROS	summer	29	0.44 ± 0.14	0.07 ± 0.09	–0.41 ± 0.08	–1.10 ± 0.33	0.74 ± 0.32	20.0 ± 7.28		–376 ± 124	1.10 ± 0.25	0.80
	winter	28	0.42 ± 0.09	0.33 ± 0.17					13.5 ± 7.22		–0.25 ± 0.11	0.77
	combined data^b^	57	0.59 ± 0.06								–0.01 ± 0.09	0.64

a Multiple linear regression equations: cellular ROS = *a* × *F*1 + *b* × *F*2 + *c* × *F*3... + intercept, where *F*1, *F*2, *F*3 (etc.) are mass concentrations of AMS-identified OA types and Fe, Cu, Mn, Cr, Zn, and a, b, c (etc.) are the corresponding coefficients with units of AUC μg^–1^.

b Combined summer and winter data. Isoprene-OA and BBOA are not identified in winter and summer, respectively, so their values in the corresponding season are necessarily set to “0” to perform the MLRM analysis.

**Figure 2:**
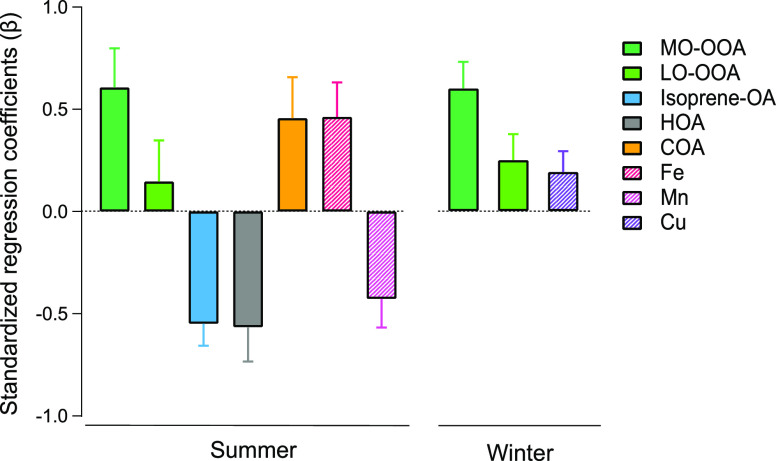
Standardized regression coefficients (β) of selected PM components for cellular ROS production. The standardized regression coefficients are derived from unstandardized regression coefficients, which are resolved from the MLRM. The corresponding MLRM results of unstandardized regression coefficients are shown in Table 1. OA types identified via AMS (MO-OOA, LO-OOA, HOA, COA, isoprene-OA, and BBOA) and five water-soluble transition metals (Fe, Cu, Mn, Cr, and Zn) are selected as independent variables for the MLRM. Only variables resolved as predictors are shown in the figure.

To compare the relative importance of the MLRM-resolved predictors for cellular ROS production that accounts for actual exposure (i.e., mass concentration), standardized regression coefficients (β) were applied, which were calculated by multiplying the unstandardized coefficients by the ratio of the standard deviations of the independent variables and dependent variables (the equation is provided in the Supporting Information).^67^ A higher absolute value of the standardized regression coefficient indicates a stronger influence of the independent variable (on the dependent variable). The results of standardized regression coefficients are shown in Figure 2. MO-OOA (β_summer_ = 0.61 ± 0.19, β_winter_ = 0.60 ± 0.13) and LO-OOA (β_summer_ = 0.15 ± 0.20, β_winter_ = 0.25 ± 0.13) are consistently selected as positive predictors for cellular ROS production for both summer and winter samples. This observation is in accord with the hierarchical cluster results, substantiating the existence of a linkage between cellular ROS production and OOA. Importantly, the β values of MO-OOA are the highest among the predictors in both seasons, indicating that it has the strongest positive effect on cellular ROS production.

COA (β_summer_ = 0.46 ± 0.20) is found to positively influence cellular ROS production in summer, yet it is not resolved as a predictor in winter. This could be because COA is more oxidized in summer than in winter (carbon oxidation state: −1.08 in summer and −1.25 in winter). Isoprene-OA (formed from the oxidation of the most abundant nonmethane hydrocarbon, isoprene) and HOA are resolved with negative regression coefficients. The negative values imply that isoprene-OA and HOA might not be responsible for the observed cellular ROS production in Atlanta. Yet, it is noted that isoprene-OA alone could induce cellular ROS production and expression of oxidative stress response genes.^29,30,68^

Previously, biomass burning aerosol collected from the Amazon has been found to induce higher levels of cellular ROS compared to PM collected in Atlanta and laboratory-generated SOA.^36^ Interestingly, while BBOA is an important OA type during winter in this study (21% of OA, Figure S2), it is not resolved as a predictor for cellular ROS production. This could be due to the highly oxidized portion of BBOA in Atlanta being apportioned to MO-OOA and thus the identified BBOA factor is mostly fresh.^23^ For instance, the *f*_44_ (fraction of the CO_2_^+^ fragment in the AMS mass spectra, indication of the oxidation level of OA) of BBOA in this study (0.06) is smaller than the reported *f*_44_ of BBOA in the Amazon, which was always >0.05 and ∼0.12 after 2 h of aging,^69^ indicating that the identified BBOA in Atlanta is relatively fresher.

Note that cellular ROS measurements were results from exposure to PM_2.5_, while AMS characterized the OA composition in PM_1_. However, our recent study at the same site shows that most OA is in PM_1_.^45^ The OA types in PM_1_ and PM_2.5_ are highly correlated with OA in PM_1_ accounting for over 80% of that in PM_2.5_.^45^ Thus, the chemical difference of OA between PM_1_ and PM_2.5_ in our study should be negligible. In this study, we have colocated AMS and PM_2.5_-ACSM (Aerosol Chemical Speciation Monitor) data for a certain period (January 20 to February 10, 2018) during the winter sampling,^45^ and we obtained consistent results from the MLRM for both AMS and PM_2.5_-ACSM data (Figure S4), showing that OOA (MO-OOA and LO-OOA) is the main contributor to cellular ROS production.

For transition metals, soluble Fe (β_summer_ = 0.46 ± 0.17) and Cu (β_winter_ = 0.19 ± 0.10) are resolved as positive predictors for cellular ROS production. Fe and Cu are the two most abundant transition metals in PM_2.5_ in the southeastern United States,^70^ but their mass concentrations are much lower than OOA (by about two orders of magnitude, Table S4). They are redox-active metals that could participate in redox cycling and Fenton-like reactions, thereby may be involved in ROS production.^10,71^ Fe and Cu are found to play an important role in cellular ROS production in different seasons. Fe is resolved as a predictor in summer while Cu is a predictor in winter. The seasonal difference in their roles in cellular ROS production should be explored in future work. In contrast, Zn is another abundant transition metal, yet it is not resolved as a predictor, likely due to its redox-inactive property.^57^ Note that the MLRM assumes that the effects of each predictor are additive, while some studies have suggested the possibility of synergistic and/or antagonistic interactions among PM components (e.g., interactions between quinones and transition metals) in ROS production.^63,72−74^ Our MLRM approach enables the first analysis to resolve the relative importance of OA types and transition metals for cellular ROS production. Yet, further research can build on these informative results to further constrain the synergistic and/or antagonistic interactions among PM components and their combined contributions to overall PM toxicity.

Overall, our results indicate that both OA and some transition metals significantly contribute to cellular ROS production. Furthermore, the results here reveal that OOA (i.e., the sum of MO-OOA and LO-OOA) is the biggest contributor to cellular ROS production among all OA types (β_summer_ = 0.76, β_winter_ = 0.85). OOA (surrogate of SOA) is largely formed from the atmospheric oxidation and aging of biogenic and anthropogenic organic gases. Pye et al.^6^ compared modeled PM_2.5_ and health effect data and reported a robust association between SOA mass concentrations and county-level cardiorespiratory death rates across the United States. Their associations are elevated in the southeastern compared to the rest of the contiguous United States. Our experimental finding here strengthens SOA in an urban setting as a major driver of PM_2.5_ in cellular ROS production, and importantly, our work quantifies the relative contribution of different SOA subtypes and demonstrates a major contribution from highly oxidized SOA (i.e., MO-OOA). As ambient OA is highly multifunctional,^25,75^ this highlights the important question of: what chemical features (e.g., structural features and functional groups) in OOA are responsible for cellular ROS production upon PM_2.5_ exposure?

### 3.3 Highly Oxidized and Aromatic Species Are Associated with Oxygenated OA and Cellular ROS Production

To unravel the chemical features of OOA contributing to cellular ROS production, we employed LC–MS/MS, FIGAERO-CIMS, and FT-IR spectrometry to characterize the molecular composition and functional groups in OA. It has been suggested that oxidized derivatives of aromatic compounds (e.g., quinone-type compounds) or conjugated double bonds (e.g., conjugated carbonyls) tend to induce cellular ROS production as they are known to participate in electron transfer reactions, which could result in ROS formation.^17,76,77^ Thus, we categorized the compounds identified from LC–MS/MS and FIGAERO-CIMS analyses into four groups for each season, according to their DU/C, i.e., DU/C = 0, 0 < DU/C < 0.25, 0.25 ≤ DU/C < 0.5, and DU/C ≥ 0.5. This metric aims to represent the number of (equivalent) double bonds (e.g., C=C, C=O, and ring structure) in each carbon atom, such that DU/C = 0.5 would indicate one double bond shared by two carbon atoms on average. The fractions of compounds of different carbon numbers in each DU/C group from LC–MS/MS and FIGAERO-CIMS analyses are shown in Figure S5. The carbon oxidation state of the compounds in each group was calculated to evaluate if the specific group of compounds contributes to AMS-identified OOA based on its carbon oxidation state.^78,79^ The calculation methods for the carbon oxidation state from LC–MS/MS data, FIGAERO-CIMS data, and AMS-identified OA types are provided in the Supporting Information.

**Figure 3:**
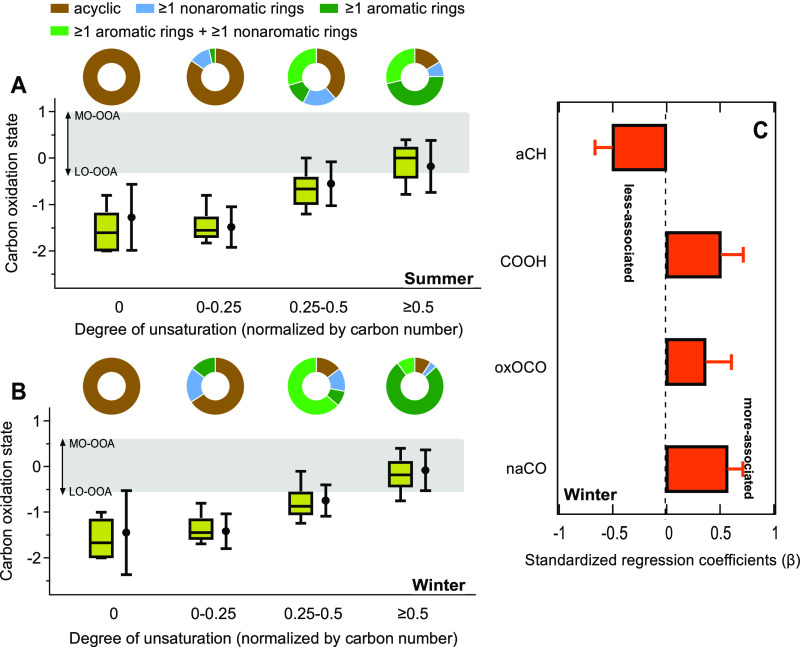
Associations between classified molecular features and functional groups with OOA. (A,B) Molecular composition and properties identified by LC–MS/MS for summer and winter periods, respectively. The same analysis results based on FIGAERO-CIMS data are shown in Figure S6. The identified compounds are categorized into four groups according to their degree of unsaturation normalized by carbon numbers (DU/C, the formula used to calculate DU/C is provided in the Supporting Information, eq S6). Each group contains 34 to 199 compounds, with DU/C = 0 having the fewest number of compounds. The box and whisker plots represent the distribution of carbon oxidation states for the compounds in each group (boxes at 25th, 50th, and 75th percentiles with whiskers at 10th and 90th percentiles). The corresponding dots and error bars are the abundance-weighted mean values and standard deviations of carbon oxidation states in each group. The upper and lower bounds of the gray area indicate the carbon oxidation state of MO-OOA and LO-OOA identified from PMF analysis of AMS data, respectively. The donut plots show the abundance-weighted contribution of acyclic compounds (brown), compounds containing ≥1 aromatic rings (dark green), ≥1 nonaromatic rings (blue), and ≥1 aromatic and nonaromatic rings (green). (C) Standardized regression coefficients of four functional groups for OOA in the winter period from FT-IR analysis. The MLRM results and unstandardized regression coefficients are displayed in Table S5.

For the LC–MS/MS data, we find that the range of the carbon oxidation state of compounds in the 0.25 ≤ DU/C < 0.5 group is similar to the carbon oxidation state of the LO-OOA observed at the site; the range of the carbon oxidation state of compounds in the DU/C ≥ 0.5 group falls into the carbon oxidation state range of OOA (bounded by LO-OOA and MO-OOA, Figure 3A,B). The carbon oxidation states of the compounds in the 0.25 ≤ DU/C < 0.5 and DU/C ≥ 0.5 groups are statistically higher than those in the DU/C = 0 and 0 < DU/C < 0.25 groups (see ANOVA results shown in Table S3). Similar results (Figure S6) were obtained using FIGAERO-CIMS data. In addition, for the top 50 compounds showing the strongest correlations with MO-OOA and LO-OOA in the FIGAERO-CIMS data, over 94% of these compounds exhibit DU/C ≥ 0.25 and a majority of the compounds (over 82%) most strongly correlated with MO-OOA have DU/C ≥ 0.5 (Figure S7). Together, these results demonstrate that highly unsaturated species are also highly oxidized and thus are associated with OOA.

It is well known that OOA is characterized by high fractions of unsaturated compounds like carboxylic acids and also nonacid oxygenates (e.g., carbonyls), based on prominent signals of *m*/*z* 44 (CO_2_^+^) and *m*/*z* 43 (C_2_H_3_O^+^) in the AMS mass spectra.^18,45,79^ OOA in the southeastern United States is largely contributed from biogenic sources and contains considerable amounts of carboxylic acids, particularly in warm seasons.^26,44,58,80^ The important contributions of carboxylic acids and carbonyls to OOA are also resolved by FT-IR analysis and the MLRM along with oxalates (Figure 3C, a detailed discussion on the FT-IR analysis and MLRM results is provided in the Supporting Information). When these functional groups with carbon–oxygen double bonds attached to a conjugated or aromatic system, such as in high DU/C compounds, the resulting compounds (e.g., quinone-type compounds and humic-like substances) can be redox-active^37,81,82^ and thus contribute to cellular ROS production. Importantly, we observe that for the compounds in the 0.25 ≤ DU/C < 0.5 and DU/C ≥ 0.5 groups in LC–MS/MS analysis, the abundance-weighted contribution of ring-containing compounds account for more than 61% (up to 91% in winter) of the identified compounds, with over 42% (up to 87% in winter) of them being aromatics (Figure 3A,B). Aromatic rings containing 5–6 carbon atoms were identified here, some of which contain a heterocyclic oxygen, nitrogen, or sulfur atom in the ring. Therefore, the carbon–oxygen double bonds determined by FT-IR and the aromatic rings identified by LC–MS/MS are likely both major functional groups of OOA contributing to cellular ROS production.

These highly oxidized and aromatic compounds in OOA are likely anthropogenic SOA, which could be formed from precursors emitted from combustion of fossil and biomass fuels.^83^ A recent study also suggests that the photochemical aging of polycyclic aromatic hydrocarbons on soot can form highly oxidized and aromatic compounds.^84^ Based on their high DU/C values, the compounds in the DU/C ≥ 0.5 group logically contain conjugated double bonds and/or aromatic structures, while the ones in the 0.25 ≤ DU/C < 0.5 group can also contain similar chemical structures. Given the high carbon oxidation states for compounds in the 0.25 ≤ DU/C < 0.5 and DU/C ≥ 0.5 groups, these highly oxidized and aromatic compounds could be a major reason that OOA contributes strongly to cellular ROS production.

## 4 Implications

In this work, we reveal that, among all OA types, OOA (surrogate of SOA) is the biggest contributor to cellular ROS production. The results imply OOA as a key OA component in cellular ROS production and thus warrant focused consideration for air quality management in reducing the health effects of PM_2.5_. These results also suggest that SOA becomes more toxic as it continues to oxidize in the atmosphere, which builds on the strong association revealed by Pye et al.^6^ that SOA has 6.5 times higher cardiorespiratory disease mortality risk than PM_2.5_ per unit mass. These consistent findings highlight the critical role of certain forms of SOA (e.g., containing carbon–oxygen double bonds and aromatic rings) and in particular highly oxidized forms of this SOA for both respiratory ROS generation and cardiovascular risks that drive the adverse health effects of PM_2.5_. While the role of oxidative stress in mediating acute health effects in the lung is compelling, its role in chronic health effects needs further exploration.^85^ Establishing the importance of oxidative stress in chronic effects can strengthen the representativeness of cellular ROS production for PM_2.5_ toxicity.

The sources, formation pathways, and chemical composition of SOA can vary vastly at different locations.^18,22^ For example, biogenic SOA is the dominant SOA in the southeastern United States, while its formation is largely influenced by anthropogenic emissions (e.g., anthropogenic NO_*x*_ and SO_2_).^44,86^ In contrast, anthropogenic SOA is more abundant than biogenic SOA in many western United States cities (e.g., Los Angeles).^87^ We have previously studied cellular ROS production of individual SOA formed from a variety of biogenic and anthropogenic precursors in laboratory experiments.^30,32^ SOA formed from aromatic precursors was found to induce higher levels of cellular ROS production. This is consistent with the finding in this work that highly oxidized and unsaturated compounds containing carbon–oxygen double bonds and aromatic rings in SOA are likely the main species responsible for cellular ROS production. Once formed, SOA continues to age in the atmosphere. Laboratory studies comparing the toxicity of aged and fresh SOA have shown a higher toxic effect of aged SOA.^27,28,31,32,88^ This is supportive to our finding here where we reveal a major contribution of MO-OOA to cellular ROS production upon exposure to ambient PM_2.5_. Much work is still needed to study and fully characterize SOA across the United States and around the world and its atmospheric aging. This, combined with more toxicological studies of SOA generated under different reaction conditions, would yield valuable insights to improve our understanding of SOA health effects.

In addition, the contribution from fossil sources to SOA formation has steadily declined in the United States over the past decades^89^ and this trend will likely continue, owing to effective strategies to control emissions from fossil sources. As a consequence, the relative importance of other sources is increasing. For example, biomass burning emission is expected to become a more important source of SOA, with an apparently increasing trend of wildfire frequency in recent years.^90,91^ One major characteristic of SOA formed from biomass burning is the high fraction of oxygenated aromatic compounds, since aromatic compounds are a major class of compounds emitted from biomass burning.^92,93^ As our work reveals that highly oxidized and unsaturated compounds containing carbon–oxygen double bonds and aromatic rings in SOA are the main species responsible for cellular ROS production, SOA may become more toxic per unit mass in the future, resulting from a larger contribution of biomass burning to SOA formation.

Future work should continue to investigate the health impacts of different PM_2.5_ components, particularly SOA formed from precursors emitted during incomplete combustion processes of fossil and biomass fuels. Given SOA’s extraordinary chemical complexity and diversity and its possible high toxicity, more detailed studies to determine what species and processes drive the SOA toxicity are needed. While this study region focuses on Atlanta across two different seasons, our work demonstrates how advanced multi-instrument chemical characterization of SOA and cellular measurements provide novel insights into the components and chemical features that drive SOA toxicity. Future studies can further leverage these strategies to advance the understanding of the health impacts of SOA and its subcomponents through collaborative research between atmospheric chemistry, toxicology, epidemiology, and biotechnology.^6,51,94^ Finally, long-term measurement of SOA is very limited^24^ and SOA is not directly considered in current epidemiological studies. Given the important role of SOA in PM_2.5_ in cellular ROS production, long-term, advanced measurements of OA types (including different types of SOA) over a wide range of geographical regions like the ongoing ASCENT (United States, https://ascent.research.gatech.edu/) and ACTRIS (Europe, www.actris.eu) networks are critical to advance the understanding of the health impacts of SOA.

## Supporting Information Available

PM chemical characterization methods; FT-IR analysis and MLRM results; representative dose–response curve; mass concentrations of nonrefractory PM_1_ and mass fractions of OA; correlation analysis; standardized regression coefficients; fractions of compounds of different carbon numbers; molecular composition and properties of OA identified by FIGAERO-CIMS; clustergrams; FIGAERO-CIMS sum thermograms; online measurements and PM sample information; tolerance and VIF; ANOVA results; mass concentrations of OA and transition metals; MLRM-resolved regression coefficients; and carbon oxidation state (PDF)
