# Supplementary material for: Oxidized and Unsaturated: Key Organic Aerosol Traits Associated with Cellular Reactive Oxygen Species Production in the Southeastern United States
Source: Environ Sci Technol. 2023 Sep 12;57(38):14150–61. doi: 10.1021/acs.est.3c03641 (PMC10538939; doi:10.1021/acs.est.3c03641)
Supplement: Supplementary file 1 — es3c03641_si_001.pdf [file es3c03641_si_001.pdf]

Supporting Information for

Oxidized and unsaturated: key organic aerosol traits associated with cellular reactive oxygen species production in the southeastern United States

Fobang Liu[a,b], Taekyu Joo[c,1], Jenna C. Ditto[d,2], Maria G. Saavedra[c,3], Masayuki Takeuchi[f], Alexandra J. Boris[e,4], Yuhan Yang[c], Rodney J. Weber[c], Ann M. Dillner[e], Drew R. Gentner[d], Nga L. Ng[b,c,f*]

[a]Department of Environmental Science and Engineering, School of Energy and Power Engineering, Xi'an Jiaotong University, Xi'an, Shaanxi 710049, China

[b]School of Chemical and Biomolecular Engineering, Georgia Institute of Technology, Atlanta, Georgia 30332, United States.

[c]School of Earth and Atmospheric Sciences, Georgia Institute of Technology, Atlanta, Georgia 30332, United States.

[d]Department of Chemical and Environmental Engineering, Yale University, New Haven, Connecticut 06511, United States.

[e]Air Quality Research Center, University of California Davis, Davis, California 95618, United States.

[f]School of Civil and Environmental Engineering, Georgia Institute of Technology, Atlanta, Georgia 30332, United States.

[*]Corresponding author: Nga L. Ng
Email: ng@chbe.gatech.edu

[1]Present address: Department of Chemical and Environmental Engineering, Yale University, New Haven, Connecticut 06511, United States

[2]Present address: Department of Energy, Environmental, and Chemical Engineering, Washington University in St. Louis, St. Louis, Missouri 63130, United States

[3]Present address: Air Quality Regulatory Consulting Services, Montrose Environmental Group, Atlanta, Georgia 30350, United States

[4]Present address: California Air Resources Board, Sacramento, California, 95811, United States

Number of pages: 27

Number of figures: 12

Number of tables: 6

1. Methods

1.1. PM chemical characterization

1.1.1 Online measurements

HR-ToF-AMS: A high-resolution time-of-flight aerosol mass spectrometer (HR-ToF-AMS, Aerodyne Research Inc.) was coupled with a $PM_{2.5}$ cyclone facing west at 4.5 m above the ground. A nafion dryer was placed upstream of the instrument to dry the particles below relative humidity (RH) of 20% in order to minimize the influence of RH on the collection efficiency (CE).[1] The composition-dependent CE correction was applied following the procedure in Middlebrook et al. [2]. Detailed description of the instrument can be found from previous studies [3-6]. Ionization efficiency (IE) calibrations were performed on a weekly basis with 300 nm ammonium nitrate and ammonium sulfate. The average sample time was set at 1 min, and the data analysis was performed in Igor Pro 6.34 (WaveMetrics Inc.) using Squirrel v1.53 and PIKA v1.12.

Positive Matrix Factorization (PMF) analysis was performed on the high-resolution organic mass spectra (*m/z* 12–150) using PMF Evaluation Toolkit (PET v.2.0x) to determine the sources of organic aerosol. PMF analysis deconvolves the organic data matrix as a linear combination of various factors with constant organic mass spectra but varying concentrations across the dataset.[7, 8] The details of preprocessing of organic data and error matrices are described in Joo et al. [3]. Five OA types were identified in summer (MO-OOA, LO-OOA, HOA, COA, and Isoprene-OA) and winter (MO-OOA, LO-OOA, HOA, COA, and BBOA), respectively.[3, 4, 9, 10] Briefly, MO-OOA and LO-OOA were characterized by dominant signals at *m/z* 44 (CO_2^+) and *m/z* 43 ($C_2H_3O^+$), with MO-OOA having a higher degree of oxidation. HOA was distinguished by alkyl fragments signatures and rush hour peaks. COA was determined by prominent signals at *m/z* 41 ($C_3H_5^+$) and *m/z* 55 ($C_4H_7^+$), and higher *m/z* 55 to 57 ratio compared to the other OA types. Isoprene-OA was characterized by dominant signals at *m/z* 53 ($C_4H_5^+$) and *m/z* 82 ($C_5H_6O^+$). BBOA was characterized by enhanced signals at *m/z* 60 ($C_2H_4O_2^+$) and *m/z* 73 ($C_3H_5O_2^+$), which are major fragment ions for levoglucosan.

Note that the instrument does not measure black carbon. While black carbon has also been suggested to play a role in adverse health effects of $PM_{2.5}$,[11-15] its role is not considered as our work is mainly determining cellular ROS production for water-soluble $PM_{2.5}$.

FIGAERO-CIMS: A filter inlet for gases and aerosols coupled to an iodide-adduct chemical ionization mass spectrometer (FIGAERO-CIMS) was deployed to measure the gas-phase and particle-phase oxidized organic compounds.[16-18]

Gas-phase measurements took place for 30 min, during which ambient aerosol was collected onto a Teflon filter (Pall). Ambient air was sampled through a 1 μm cyclone (URG) at 16.7 L/min, and was then subsampled at ~6.8 L/min through the filter for aerosol collection. After 30-min collection, the filter port was moved to the CIMS inlet and heated dry N_2 gas flowed through the filter to vaporize collected aerosols on the filter. Vaporized compounds were then carried into the CIMS for measurements. The desorption period lasted for 30 min; temperature of N_2 gas increased linearly from ~30 to 200 °C for 15 min during the ramping stage, stayed at 200 °C for 10 min during the soaking stage, and cooled back to 30 °C for 5 min during the cooling stage. The sum thermograms during summer and winter sampling are provided in Figure S10. In total, one complete measurement cycle took place every hour. Once every 7 cycles, ambient air was sampled through an additional filter prior to the filter collection to serve as particle-phase background measurements. Raw signal data were pre-averaged to 10-s data and were further analyzed using Tofware v2.5.11 in Igor Pro 6.[19] Signals for particle-phase measurements were integrated area during the desorption period with background subtraction.

1.1.2 Offline measurements

LC-MS/MS analysis: The procedures of LC-MS/MS analysis are as follows: for liquid chromatography, Milli-Q water with 0.1% acetic acid (A) and methanol (B) at 0.2 mL/min were used as mobile phases with an Agilent Poroshell 120 SQ-Aq reverse phase column (2.1×50 mm, 2.7 μm particle size). The following solvent gradient was used: from 0 to 2 min, 95% A and 5% B; from 2 to 22 min, increase B to 90%; from 22 to 27 min, hold at 90% B; then decrease to 5% B to prepare for the next run. Electrospray source parameters were set to the following: drying gas temperature of 225 °C and flow of 17 L/min, fragmentor voltage of 365 V, capillary voltage of 4000 V, sheath gas temperature of 400 °C and flow of 12 L/min, nebulizer pressure at 20 psig. All ions from initial sample runs without MS/MS were investigated with MS/MS, at collision energies of 5, 10, 20, 30, and 40 V.

MS/MS spectra were analyzed with SIRIUS and CSI:FingerID [20, 21] to identify molecular structural features and functional groups. Finally, we used the APRL Substructure Search program [22] to enumerate atmospherically-relevant functional groups from the top scoring candidates exported from SIRIUS. Further methods details, including QA/QC, are described in Ditto et al. [23].

FT-IR spectrometry analysis: The collected filters were analyzed using a Bruker Tensor II FT-IR spectrometer (Bruker Optics, Inc.), operated in transmission mode, with a liquid-nitrogen-cooled mercury cadmium telluride detector. The custom-built sample chamber within the

spectrometer[24] was flushed continuously with air scrubbed of H_2O and CO_2 (model VCDA air purge system, Puregas, LLC, <10 % RH). Spectra included the wavenumber range of 4000 to 1500 cm^{-1}. Additional details about the FT-IR spectrometry analysis have been described in Takahama et al. [25]. Functional group concentrations were quantified in the ambient samples by applying multivariate regression coefficients, which were developed previously [26], to each of the sample FT-IR spectra. The quantified functional groups included aliphatic carbon (aCH), carboxylic acids (COOH), oxalates (oxOCO, representing carboxylates), non-acid and non-oxalate carbonyls (naCO), and alcohols (aCOH). Each sample functional group mass was normalized by the volume of air collected during its 8-hr or 24-hr sample period. The method detection limits (MDLs) were calculated as the 95th percentile minus the median of all blank filter functional group quantities, following the Interagency Monitoring of Protected of Visual Environments (IMPROVE) network Standard Operating Procedure 351.

1.2. Calculation of standardized regression coefficients (β) of MLRM-resolved predictors

The following equation was used to calculate standardized regression coefficients (β) of MLRM-resolved predictors:

$$\beta_i = \hat{\beta}_i(S_i/S_y) \qquad \text{(S1)}$$

where β_i and $\hat{\beta}_i$ are the standardized regression coefficient and unstandardized regression coefficient of predictor i, respectively; S_i is the standard deviation of the MLRM-input data of predictor i; S_y is the standard deviation of the MLRM-input data of the dependent variable, i.e., cellular ROS in this work.

1.3. Calculation methods of carbon oxidation state from LC-MS/MS data, FIGAERO-CIMS data, and AMS-identified OA factors

C, H, O, N, and S are the possible elements in the molecules or fragments identified by the mass spectrometry techniques used in this study. In general, to accurately calculate carbon oxidation state (OS_C), we used the formula $OS_C = 2\times(O/C) - 1\times(H/C) - a\times(N/C) - b\times(S/C)$, where a and b are the oxidation states of N and S, respectively, building off of common approaches using O/C and H/C in the literature.[27]

1.3.1. LC-MS/MS data

The LC-MS/MS analysis yielded information on both molecular formula and functional groups for the identified compounds [23]. Thus, the OS_C of an individual compound was calculated based on the identified N-containing and S-containing functional groups. If there were > 1 types of

N-containing or S-containing functional groups, an averaged N or S oxidation state was used. For example, if 1 amine (OS_C of -3) and 1 organic nitrate (OS_C of +5) group were in a compound, the overall oxidation state would be +1 for N. If N-containing or S-containing functional groups were not identified in some compounds, an oxidation state of 0 was used (for N or S). Note that the compounds with unidentified N-containing or S-containing functional groups account for < 14% of the total compounds. Thus, the OS_C results shown in Figure 3 are not affected much by these compounds.

The abundance-weighted mean value of OS_C ($\bar{x}$) was calculated using the following equation:

$$\bar{x} = \frac{\sum_{i=1}^{n} w_i x_i}{\sum_{i=1}^{n} w_i} \tag{S2}$$

where n is the number of identified compounds to be averaged, and w_i and x_i is the abundance and OS_C of i compound, respectively.

The standard deviation of the abundance-weighted average OS_C (SD_w) was calculated as follows:

$$SD_w = \sqrt{\frac{\sum_{i=1}^{n} w_i (x_i - \bar{x})^2}{\frac{n-1}{n} \sum_{i=1}^{n} w_i}} \tag{S3}$$

1.3.2. FIGAERO-CIMS data

The FIGAERO-CIMS yielded molecular formulas for each identified compound but without functional group information. To calculate the OS_C of each compound, we used a lower bound of N oxidation state of -0.7 and S oxidation state of +2.3, computed by taking the average oxidation state of all the LC-MS/MS identified compounds containing N or S, respectively. The highest possible oxidation state of N and S, i.e., +5 and +6, were used as the upper bound. This is based on both positive and negative modes of ionization were used in LC-MS/MS and N and S with negative oxidation states were more likely detected in positive mode. But only negative mode of ionization (iodide ionization method) was used in CIMS. Thus, the averaged N and S oxidation states obtained from LC-MS/MS results would be reasonable as the lower bound to calculate OS_C for FIGAERO-CIMS data. Thus, the following equations were used to calculate OS_C of individual compounds in FIGAERO-CIMS data:

$$\text{For lower bound: } OS_C = 2\times(O/C) - 1\times(H/C) + 0.7\times(N/C) - 2.3\times(S/C) \tag{S4}$$

$$\text{For upper bound: } OS_C = 2\times(O/C) - 1\times(H/C) - 5\times(N/C) - 6\times(S/C) \tag{S5}$$

The abundance-weighted mean values and standard deviation of OS_C were calculated using equation (S2) and (S3). Figures S6 (A–B) and S6 (C–D) are the lower bound and upper bound OS_C results, respectively.

Note that the OS_C values for LC-MS/MS and FIGAERO-CIMS data were calculated for four groups of identified compounds according to their degree of unsaturation normalized by carbon number (DU/C). For a formula $C_xH_yO_zN_iS_j$, DU/C was calculated as following:

$$DU/C = (2x + 2 - y + i)/2x \quad \text{(S6)}$$

1.3.3. AMS-identified OA types

Bulk O/C, H/C, N/C, and S/C were determined for the AMS-identified OA types. Then, we used equations (S4) and (S5) to calculate the lower and upper bounds of OS_C for each OA type, respectively. For comparison, we also included the results of $OS_C = 2\times(O/C) - 1\times(H/C)$, which is widely used to estimate OS_C of OA [27]. The results of OS_C from each calculation method is provided in Table S6. Note that, to be consistent with LC-MS/MS and FIGAERO-CIMS data, the OS_C results from equation (S4) were used for MO-OOA and LO-OOA in Figures 3 and S6 (A–B), and the OS_C results from equation (S5) were used in Figure S6 (C–D).

2. FT-IR analysis and MLRM results

We employed FT-IR spectrometry to measure organic functional groups for the wintertime $PM_{2.5}$ samples. The quantified functional groups include aliphatic carbon (aCH), carboxylic acids (COOH), oxalates (oxOCO, representing carboxylates), non-acid and non-oxalate carbonyls (naCO), and alcohols (aCOH). The mass concentrations of functional groups measured by FT-IR account for 79% of OA mass concentration measured by AMS (Figure S11), demonstrating these are dominant functional groups in OA.

We used MLRM to gain quantitative insights into the contribution of each functional group to OOA (Table S5, Figure 3C; results for all AMS-identified OA types and total AMS OA are shown in Figure S12). The contributions from aCH, COOH, oxOCO, and naCO are captured by the model, where positive regression coefficients are found for COOH, oxOCO, and naCO, and a negative regression coefficient is found for aCH. The negative association between aCH and OOA could reflect that the relative abundance of aCH would decrease when more OA is in the form of OOA.

Additional functional groups such as aromatics, organonitrates, and peroxides are not included in the MLRM analysis because FT-IR calibrations to measure these functional groups have not been developed. Measurements of these functional groups on filter samples using FT-IR have been proved challenging due to the low absorbance and varying peak shapes of aromatics [28], stability of

peroxides,[29] and stability and overlapping peaks for organonitrates.[30] The unidentified functional groups by FT-IR may also contribute to OOA, which should be further explored in future work.

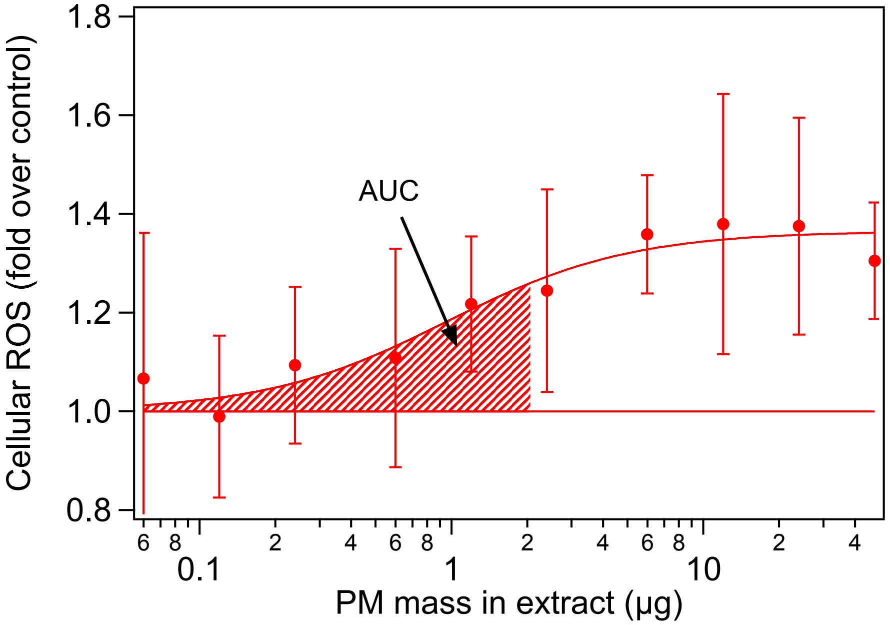

Figure S1. Representative dose response curve of ROS produced upon exposure to ambient $PM_{2.5}$ (sampling date: 2018-02-07). ROS is expressed as fold increase over control cells, defined as probe-treated cells incubated with stimulant-free media. Dose is expressed as mass in extract (µg). Data shown are means ± standard error of triplicate exposure experiments. The Hill equation was used to fit the dose-response curve and the area under the dose response curve (AUC) is shaded.

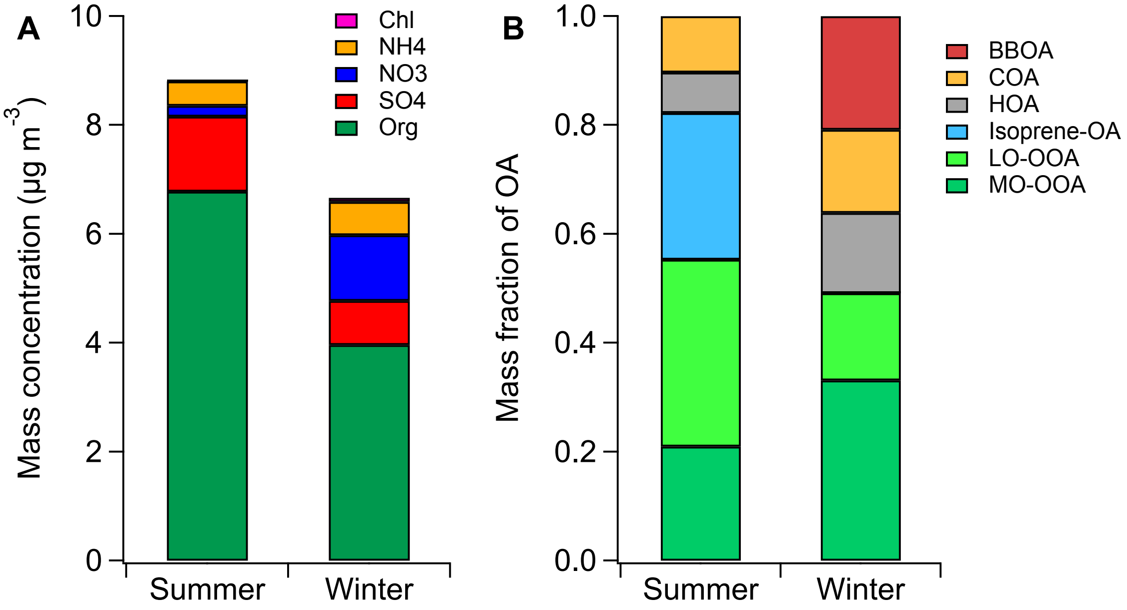

Figure S2. (A) Mass concentrations of non-refractory PM_1 species measured by HR-ToF-AMS. (B) Mass fractions of OA types identified from PMF analysis of AMS data.

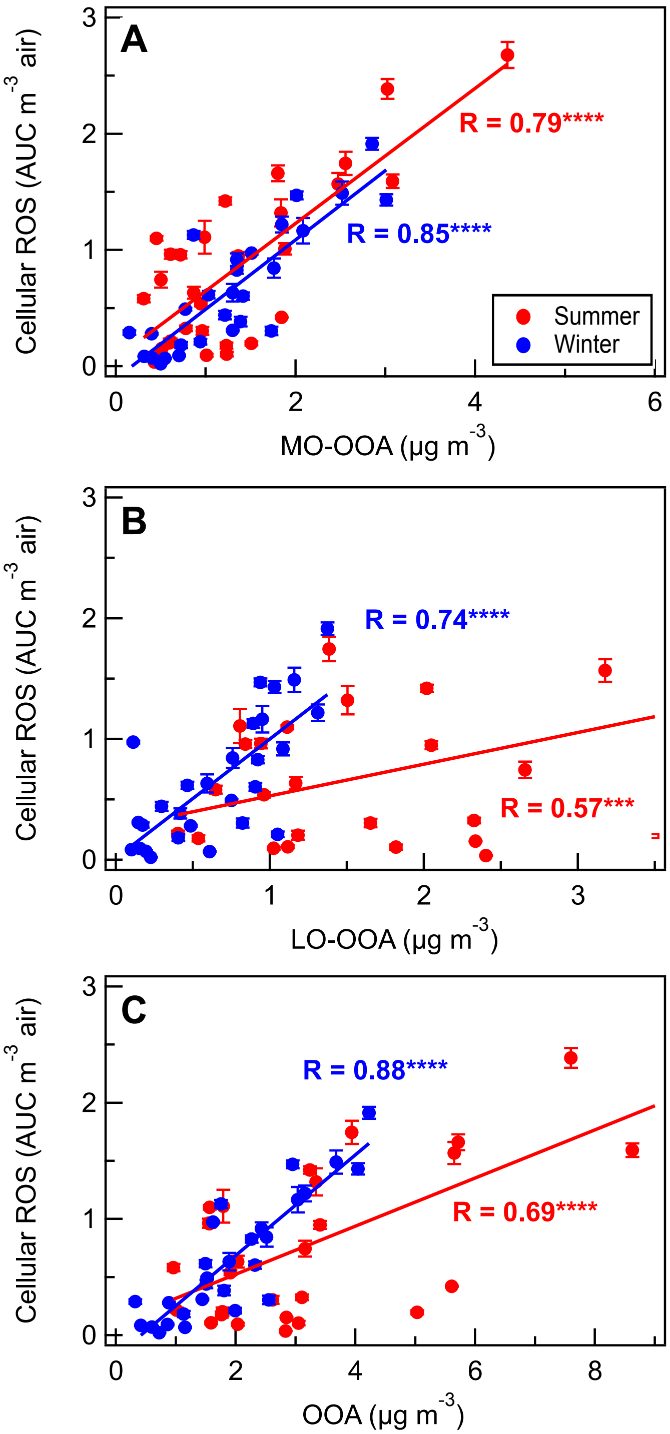

Figure S3. Correlations between cellular ROS production and mass concentrations of MO-OOA, LO-OOA, and OOA (sum of MO-OOA and LO-OOA). The error bars of cellular ROS are standard errors which are determined by fitting three dose-dependent curves through randomly removing one dose datapoint.[31] *** and **** indicate significance of $p < 0.001$ and $p < 0.0001$, respectively.

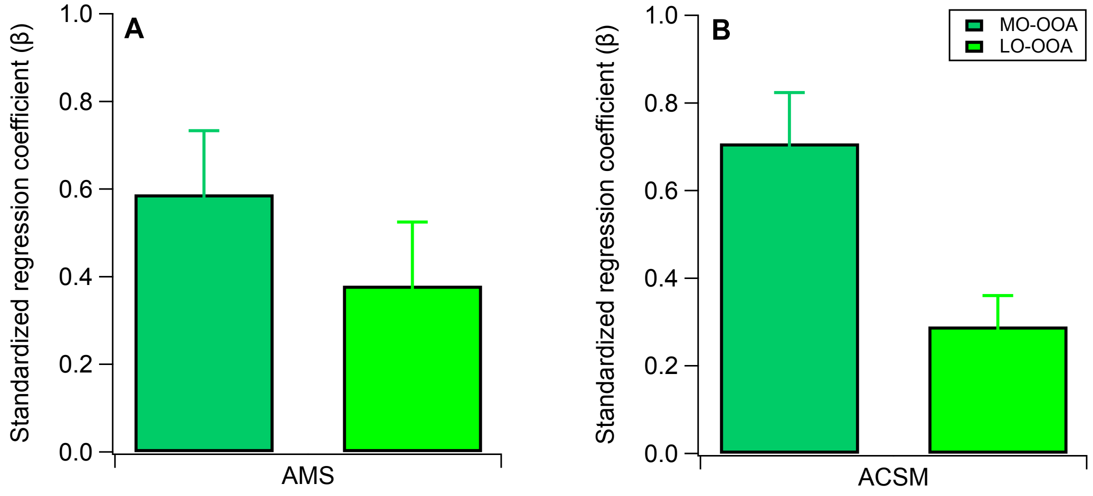

Figure S4. Standardized regression coefficients from MLRM analysis by using AMS-identified OA (A) and ACSM-identified OA (B). The AMS and ACSM data are collected from the same sampling site and the same period.

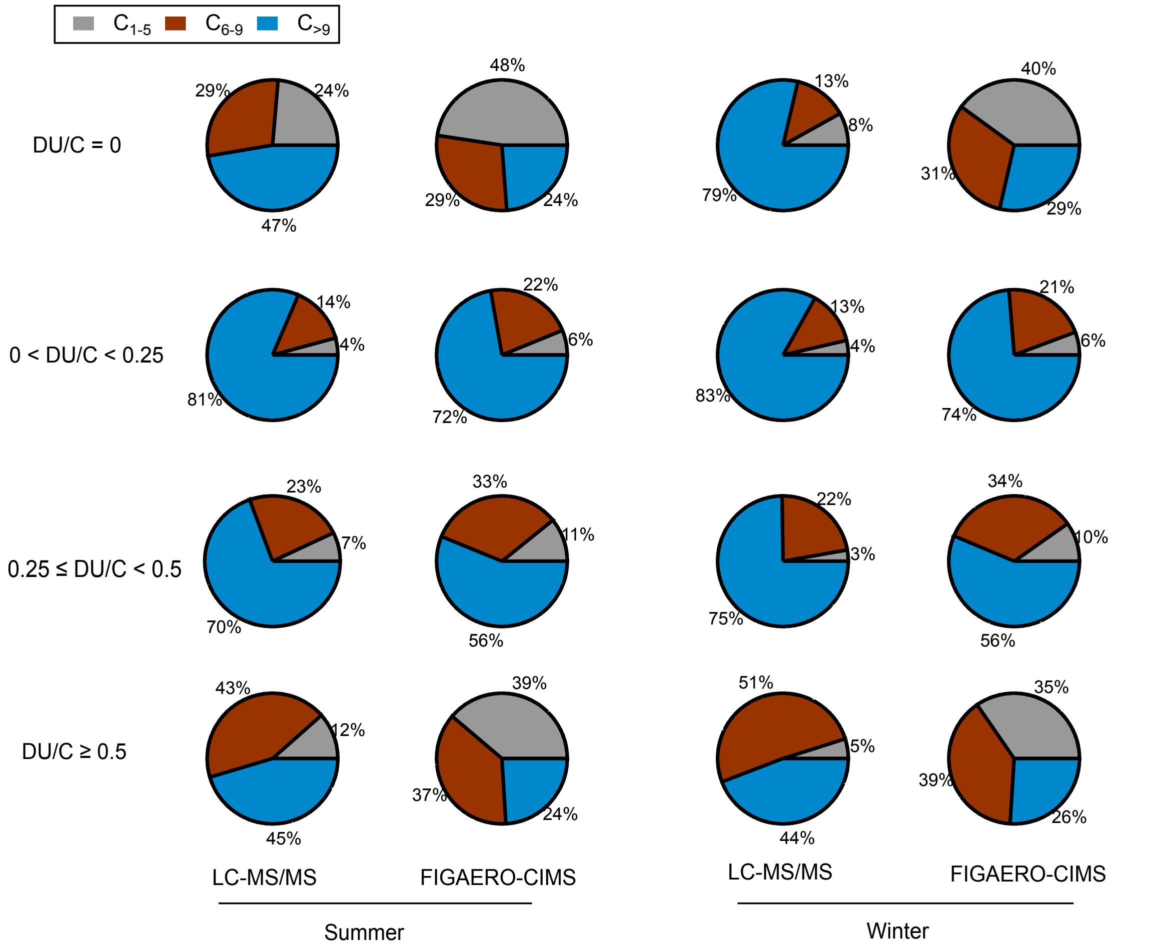

Figure S5. The fractions of compounds of different carbon numbers in each DU/C group from LC-MS/MS and FIGAERO-CIMS analyses. The LC-MS/MS and FIGAERO-CIMS employ different ionization methods (Materials and Methods), whose sensitivity varies across compound types. This is also reflected by differences of carbon number fractions in the DU/C = 0 group, i.e., $C_{>9}$ compounds are dominant in LC-MS/MS data while C_{1-5} compounds exhibit the highest fraction in FIGAERO-CIMS data. However, both techniques show the highest fraction of $C_{>9}$ compounds in the $0 < DU/C < 0.25$ and $0.25 \leq DU/C < 0.5$ groups, and a larger contribution of $C_{\leq 9}$ compounds in the $DU/C \geq 0.5$ group. (DU/C: degree of unsaturation normalized by carbon number).

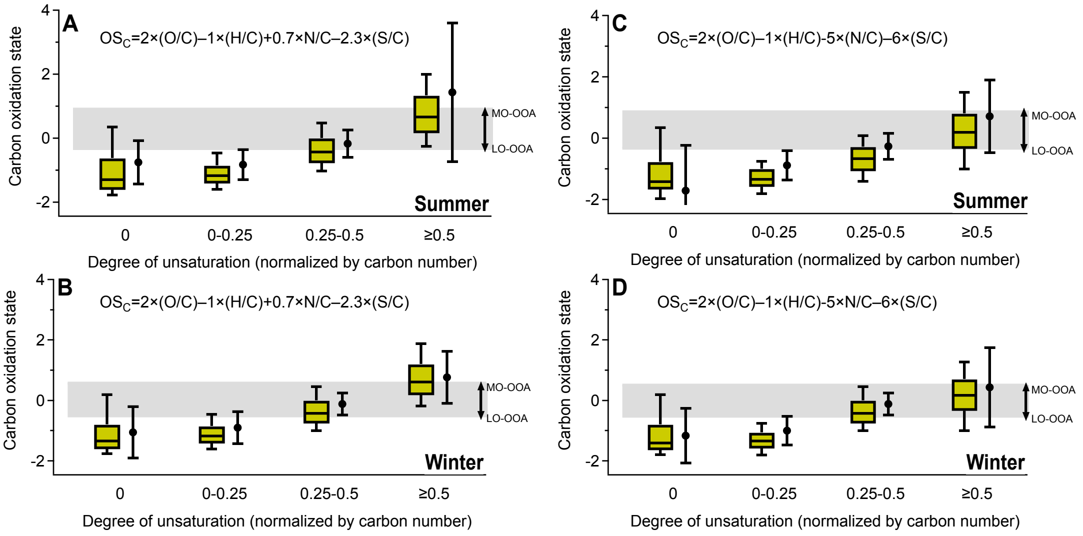

Figure S6. Molecular composition and properties of OA identified by FIGAERO-CIMS. The identified compounds were categorized into four groups according to their degree of unsaturation normalized by carbon number (DU/C). The box and whisker plots represent the range of carbon oxidation states (OS_C) for the compounds in each group. The boxes represent the 25th (lower edge), median (solid line), and 75th percentile (upper edge). The whiskers represent the 10th and 90th percentiles. The dots and error bars are the abundance-weighted mean values and standard deviations of carbon oxidation state in each group. The upper and lower bounds of the gray area indicate the carbon oxidation state of MO-OOA and LO-OOA identified from PMF analysis of AMS data, respectively. A and B are the OS_C results calculated using equation (S4), and C and D are the OS_C results calculated using equation (S5).

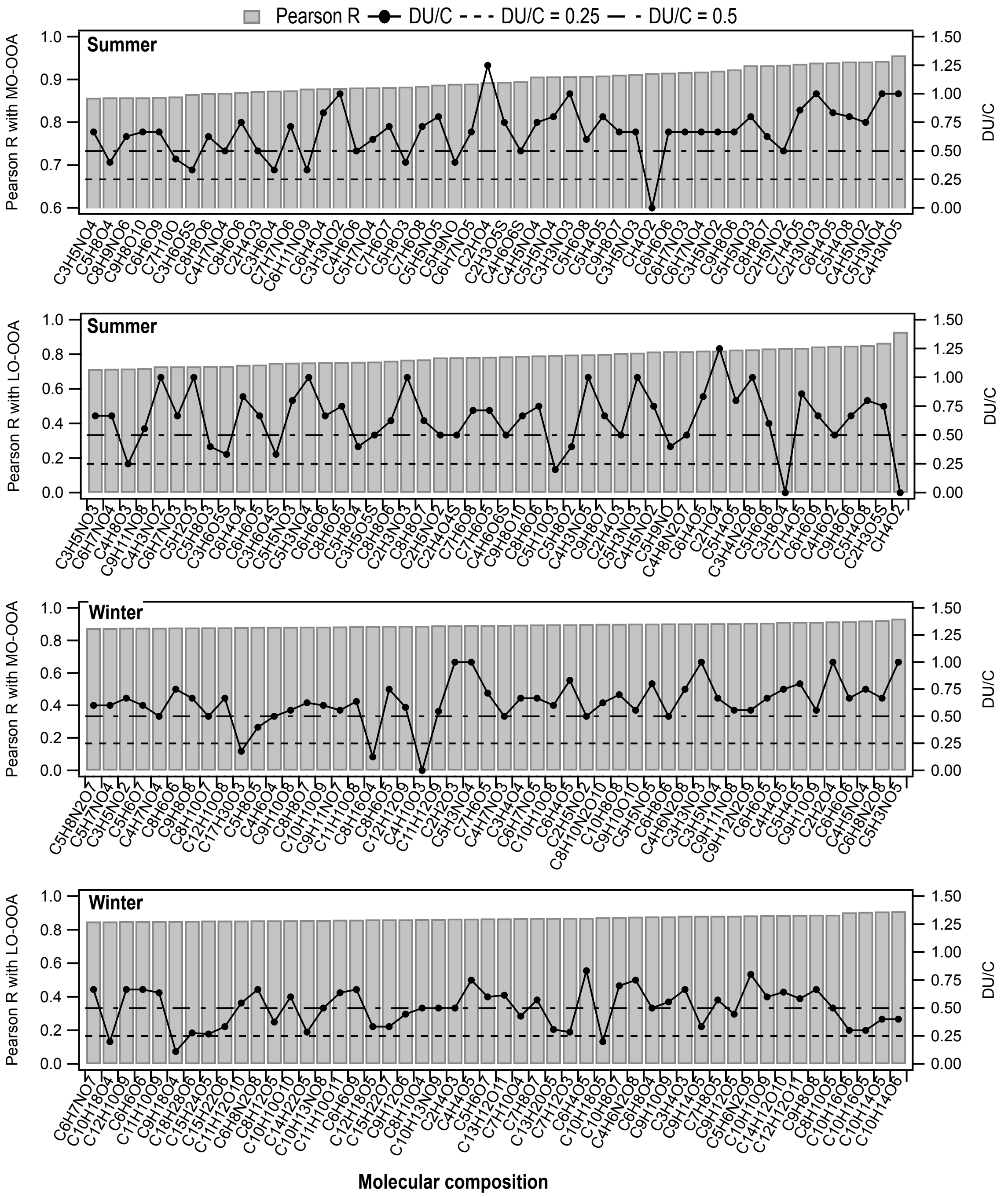

Figure S7. Degree of unsaturation normalized by carbon number (DU/C) values for the top fifty compounds most strongly correlated with MO-OOA and LO-OOA from the FIGAERO-CIMS data. Data are rank ordered by Pearson R with demarcations for DU/C values for 0.25 and 0.5.

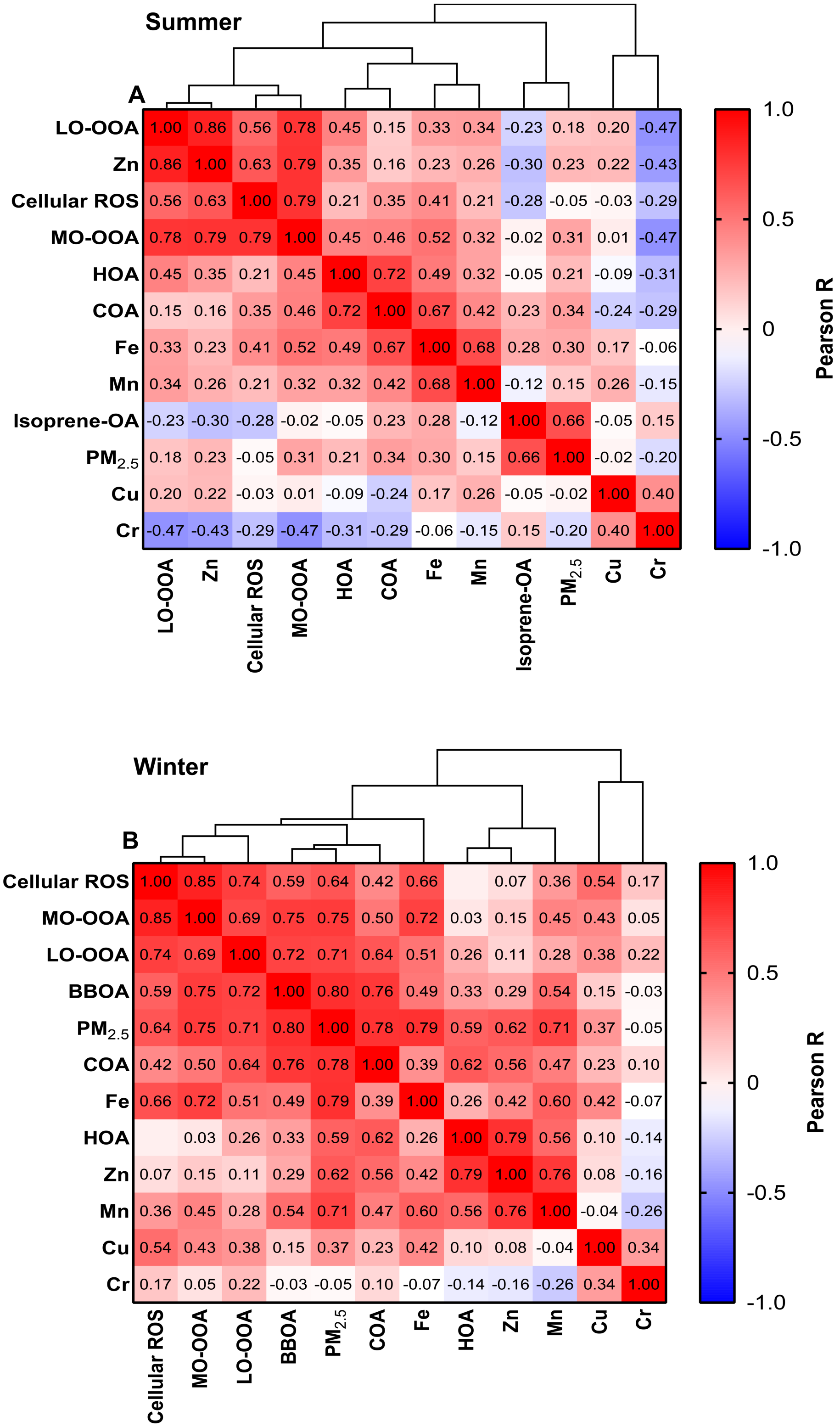

Figure S8. Clustergrams of cellular ROS and selected PM components. Summer (A) and winter (B). This figure is a duplicate version of Figure 1 but with R values on the squares.

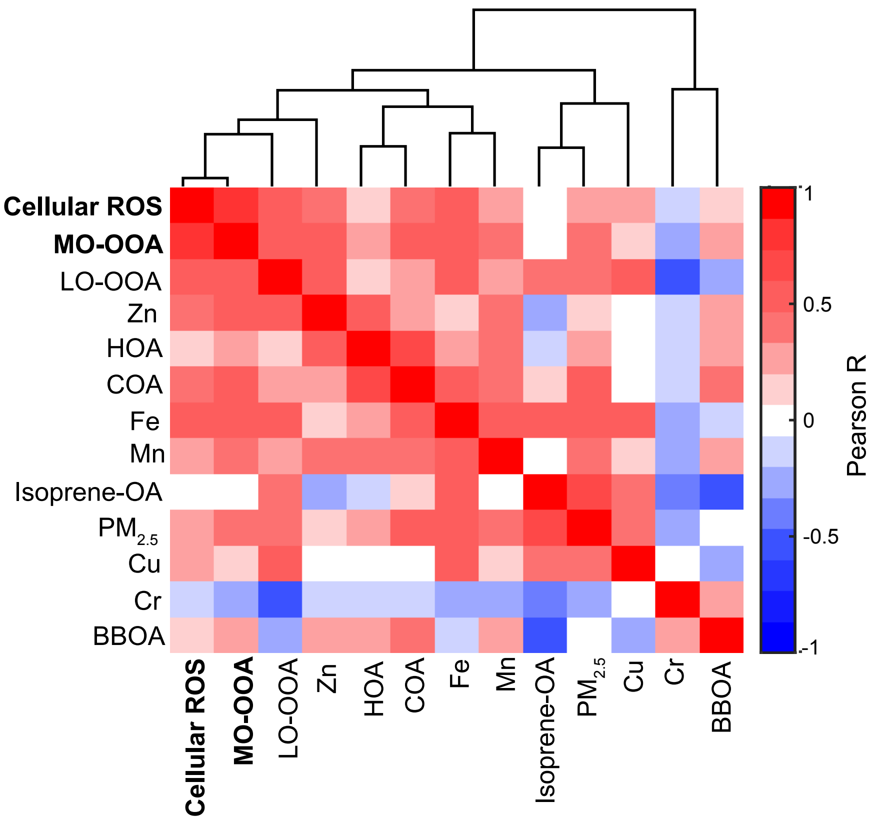

Figure S9. Clustergram of cellular ROS and selected PM components using the combined data from summer and winter. Isoprene-OA and BBOA are not identified in winter and summer, respectively, so their values in the corresponding season are necessarily set to "0" to perform the combined multi-season cluster analysis.

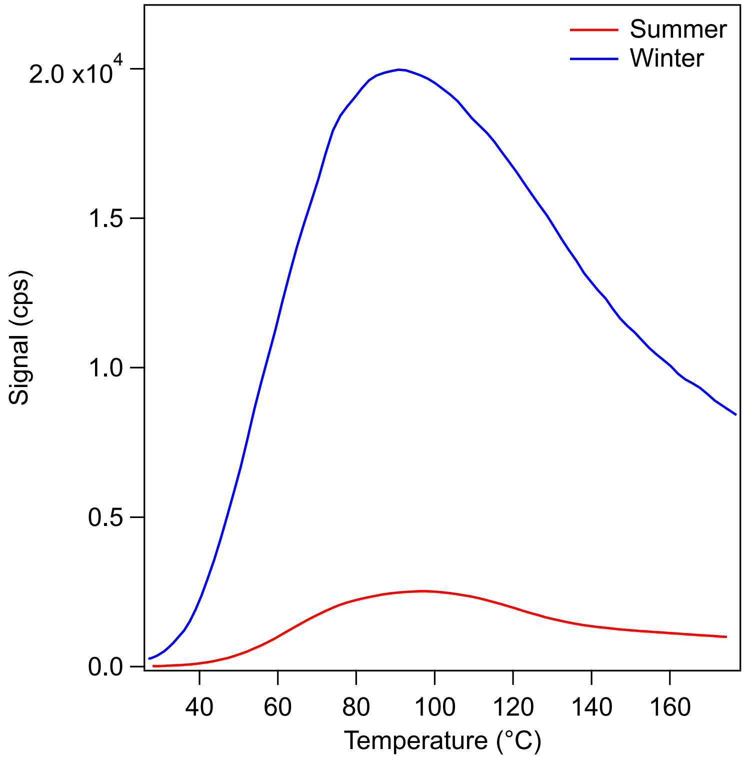

Figure S10. The FIGAERO-CIMS sum thermograms during summer and winter sampling.

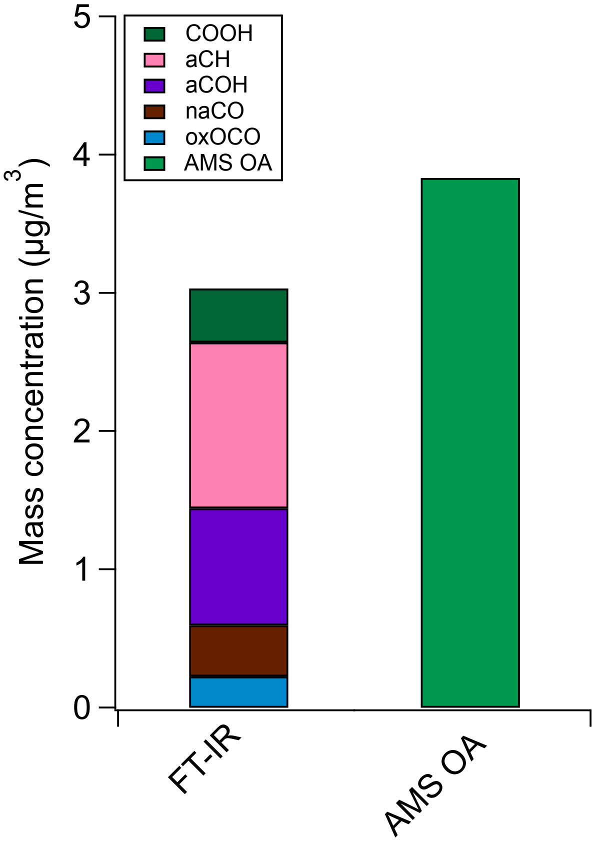

Figure S11. Mass concentrations of OA analyzed by FT-IR spectrometry (stacked by identified functional groups) and AMS (averaged OA mass concentration).

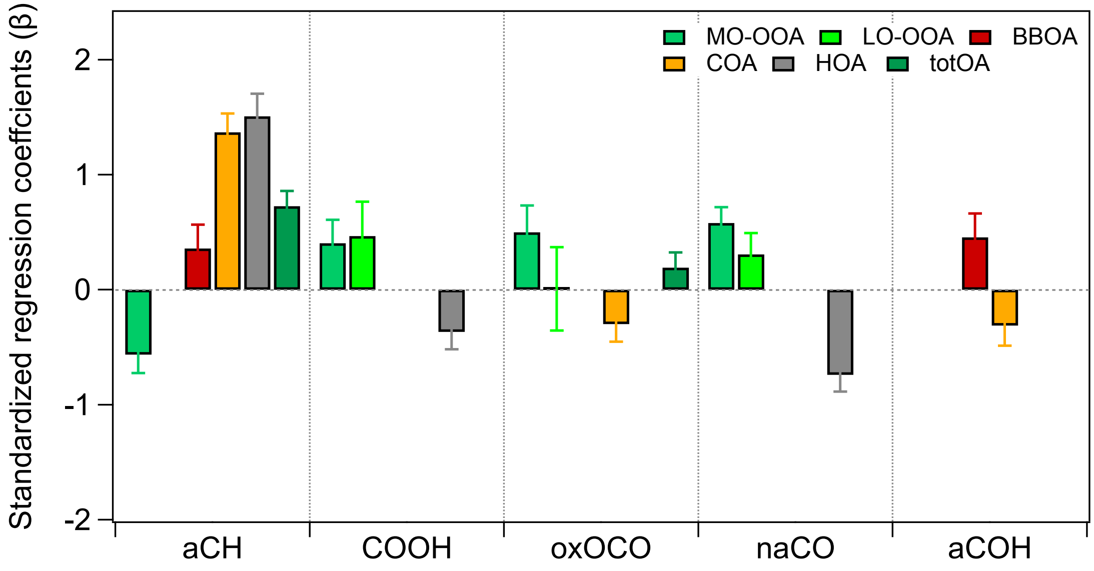

Figure S12. Standardized regression coefficients of five functional groups for AMS-identified OA types and total AMS OA in winter data. The MLRM results and unstandardized regression coefficients are displayed in Table S6. The five functional groups are measured by FT-IR spectrometry.

Table S1. Summary of online measurements and offline filter sampling.

Online measurements		
Online technique	Sampling period	
AMS	Summer: 07/19/2017 – 08/25/2017<br>Winter: 01/15/2018 – 02/11/2018	
FIGAERO-CIMS	Summer: 07/30/2017 – 08/24/2017<br>Winter: 01/20/2018 – 1/28/2018 and 2/06/2018 – 02/11/2018	
ACSM	Winter: 01/20/2018 – 02/10/2018	
**$PM_{2.5}$ samples for cellular ROS measurement and water-soluble transition metals analysis**		
Sampling period	Sampling time	Sampling size
07/19/2017 – 08/25/2017	24-hr (0:00–0:00)	29
01/15/2018 – 02/11/2018	24-hr (0:00–0:00)	28
**PM_{10} samples for LC-MS/MS analysis**		
Sampling period	Sampling time	Sampling size
07/28/2017 – 08/18/2017	Daytime (10 hr, 9:00–19:00)<br>Nighttime (6.5 hr, 22:30–5:00)	Daytime (12)<br>Nighttime (10)
01/15/2018 – 02/09/2018	Daytime (8 hr, 9:00–17:00)<br>Nighttime (8 hr, 21:00–5:00)<br>24 hr (9:00–8:55)	Daytime (14)<br>Nighttime (14)<br>24 hr (6)
**PM_1 samples for FT-IR analysis**		
Sampling period	Sampling time	Sampling size
01/22/2018 – 02/04/2018	Daytime (8 hr, 9:00–17:00)<br>Nighttime (8 hr, 21:00–5:00)<br>24 hr (9:00–8:55)	Daytime (14)<br>Nighttime (12)<br>24 hr (14)

Table S2. The output values of the tolerance and variance inflation factor (VIF) of the MLRM-resolved predictors.

(1) MLRM for cellular ROS production

	Tolerance	VIF
**Summer**		
MO-OOA	0.20	5.06
LO-OOA	0.18	5.57
Isoprene-OA	0.62	1.61
HOA	0.26	3.91
COA	0.18	5.53
Fe	0.26	3.90
Mn	0.37	2.72
**Winter**		
MO-OOA	0.49	2.04
LO-OOA	0.51	1.95
Cu	0.81	1.24

(2) MLRM for OOA, AMS-identified OA types, and total AMS OA during winter

	Tolerance	VIF
**OOA (sum of MO-OOA and LO-OOA)**		
aCH	0.23	4.43

COOH	0.14	7.09
oxOCO	0.11	9.24
naCO	0.31	3.24
**MO-OOA**		
aCH	0.23	4.43
COOH	0.14	7.09
oxOCO	0.11	9.24
naCO	0.31	3.24
**LO-OOA**		
COOH	0.16	6.26
oxOCO	0.11	9.17
naCO	0.41	2.41
**BBOA**		
aCH	0.27	4.22
aCOH	0.27	4.22
**COA**		
aCH	0.21	4.74
oxOCO	0.24	4.21
aCOH	0.18	5.50
**HOA**		
aCH	0.23	4.40
COOH	0.38	2.66
naCO	0.41	2.44
**Total AMS OA**		
aCH	0.31	3.23
oxOCO	0.31	3.23

Table S3. The ANOVA results of OSc of compounds in each DU/C group.

**Compounds identified from LC-MS/MS analysis**		
Comparisons	*p* value	
	summer	winter
DU/C = 0 vs. 0 < DU/C < 0.25	0.3367	>0.9999
DU/C = 0 vs. 0.25 ≤ DU/C < 0.5	<0.0001	<0.0001
DU/C = 0 vs. DU/C ≥ 0.5	<0.0001	<0.0001
0 < DU/C < 0.25 vs. 0.25 ≤ DU/C < 0.5	<0.0001	<0.0001
0 < DU/C < 0.25 vs. DU/C ≥ 0.5	<0.0001	<0.0001
0.25 ≤ DU/C < 0.5 vs. DU/C ≥ 0.5	<0.0001	<0.0001
**Compounds identified from FIGAERO-CIMS analysis (OSc = 2×(O/C) - 1×(H/C) + 0.7×(N/C) - 2.3×(S/C))**		
Comparisons	summer	winter
DU/C = 0 vs. 0 < DU/C < 0.25	>0.9999	>0.9999
DU/C = 0 vs. 0.25 ≤ DU/C < 0.5	<0.0001	<0.0001
DU/C = 0 vs. DU/C ≥ 0.5	<0.0001	<0.0001
0 < DU/C < 0.25 vs. 0.25 ≤ DU/C < 0.5	<0.0001	<0.0001
0 < DU/C < 0.25 vs. DU/C ≥ 0.5	<0.0001	<0.0001
0.25 ≤ DU/C < 0.5 vs. DU/C ≥ 0.5	<0.0001	<0.0001
**Compounds identified from FIGAERO-CIMS analysis (OSc = 2×(O/C) - 1×(H/C) - 5×(N/C) - 6×(S/C))**		
Comparisons	summer	winter

DU/C = 0 vs. 0 < DU/C < 0.25	>0.9999	>0.9999
DU/C = 0 vs. 0.25 ≤ DU/C < 0.5	0.0010	<0.0001
DU/C = 0 vs. DU/C ≥ 0.5	<0.0001	<0.0001
0 < DU/C < 0.25 vs. 0.25 ≤ DU/C < 0.5	<0.0001	<0.0001
0 < DU/C < 0.25 vs. DU/C ≥ 0.5	<0.0001	<0.0001
0.25 ≤ DU/C < 0.5 vs. DU/C ≥ 0.5	<0.0001	<0.0001

DU/C: degree of unsaturation normalized by carbon number

Table S4. Mass concentrations of AMS-identified OA types and five transition metals (Fe, Cu, Mn, Cr, and Zn).

μg/m^3	MO-OOA	LO-OOA	Isoprene-OA	HOA	COA	BBOA	Fe	Cu	Mn	Cr	Zn
Summer	1.34 ± 0.96	2.20 ± 1.54	1.72 ± 0.95	0.66 ± 0.43	0.48 ± 0.36		(2.54 ± 1.65) × 10^{-2}	(2.30 ± 0.94) × 10^{-2}	(2.00 ± 0.79) × 10^{-3}	(3.87 ± 2.39) × 10^{-4}	(4.31 ± 7.49) × 10^{-3}
Winter	1.28 ± 0.75	0.65 ± 0.39		0.57 ± 0.55	0.57 ± 0.43	0.82 ± 0.69	(1.08 ± 0.80) × 10^{-2}	(1.14 ± 0.74) × 10^{-2}	(1.81 ± 0.98) × 10^{-3}	(6.62 ± 2.26) × 10^{-4}	(7.12 ± 4.05) × 10^{-3}

Table S5. The MLRM-resolved regression coefficients and standard deviations of each independent variable for OOA, AMS-identified OA types, and total AMS OA during winter.

	aCH	COOH	oxOCO	naCO	aCOH	Intercept	R^2
OOA (sum of MO-OOA and LO-OOA)	-0.71 ± 0.24	1.50 ± 0.62	3.67 ± 2.39	2.68 ± 0.67		0.35 ± 0.26	0.76
MO-OOA	-0.55 ± 0.16	0.82 ± 0.41	3.43 ± 1.59	1.87 ± 0.44		0.13 ± 0.18	0.77
LO-OOA		0.55 ± 0.35	0.03 ± 1.43	0.57 ± 0.35		0.20 ± 0.16	0.44
BBOA	0.39 ± 0.23				0.72 ± 0.33	-0.30 ± 0.17	0.60
COA	1.10 ± 0.13		-1.68± 0.86		-0.36 ± 0.20	-0.04 ± 0.11	0.78
HOA	1.30 ± 0.17	-0.66 ± 0.27		-2.11 ± 0.42		0.02 ± 0.12	0.66
Total AMS OA	2.46 ± 0.45		4.58 ± 3.12			-0.15 ± 0.44	0.79

Multiple linear regression equations: OOA, or AMS-identified OA types, or total AMS OA = a×F1 + b×F2 + c×F3… + intercept, where F1, F2, F3 (etc.) are mass concentrations of the five functional groups measured by FT-IR spectrometry, and a, b, c (etc.) are the corresponding coefficients with arbitrary units.

Table S6. The carbon oxidation state (OS_C) of AMS-identified OA types.

Summer	O/C	H/C	N/C	S/C	OS_C [a]	OS_C [b]	OS_C [c]
MO-OOA	0.99	1.03	7.6e-03	3.8e-05	0.95	0.91	0.95
LO-OOA	0.50	1.33	1.9e-03	5.2e-05	-0.32	-0.33	-0.32
Isoprene-OA	0.52	1.33	2.1e-04	5.9e-05	-0.29	-0.29	-0.29
COA	0.22	1.52	3e-03	4.3e-05	-1.07	-1.09	-1.08
HOA	0.12	1.77	8.3e-03	8.6e-06	-1.51	-1.56	-1.52
Winter	O/C	H/C	N/C	S/C	OS_C [a]	OS_C [b]	OS_C [c]
MO-OOA	0.85	1.13	0.01	2.4e-04	0.58	0.51	0.57
LO-OOA	0.41	1.37	1.8e-03	1.8e-03	-0.55	-0.57	-0.55
BBOA	0.40	1.40	8.7e-03	7e-05	-0.59	-0.64	-0.60
COA	0.18	1.61	5.1e-03	4.7E-09	-1.25	-1.28	-1.25
HOA	0.11	1.79	8.4e-03	2.5e-04	-1.56	-1.61	-1.57

[a]: $OS_C = 2\times(O/C) - 1\times(H/C) + 0.7\times(N/C) - 2.3\times(S/C)$
[b]: $OS_C = 2\times(O/C) - 1\times(H/C) - 5\times(N/C) - 6\times(S/C)$
[c]: $OS_C = 2\times(O/C) - 1\times(H/C)$

References

1. Matthew, B. M.; Middlebrook, A. M.; Onasch, T. B., Collection efficiencies in an Aerodyne Aerosol Mass Spectrometer as a function of particle phase for laboratory generated aerosols. *Aerosol Science and Technology* **2008,** *42*, (11), 884-898.
2. Middlebrook, A. M.; Bahreini, R.; Jimenez, J. L.; Canagaratna, M. R., Evaluation of composition-dependent collection efficiencies for the aerodyne aerosol mass spectrometer using field data. *Aerosol Science and Technology* **2012,** *46*, (3), 258-271.
3. Joo, T.; Chen, Y.; Xu, W.; Croteau, P.; Canagaratna, M. R.; Gao, D.; Guo, H.; Saavedra, G.; Kim, S. S.; Sun, Y., Evaluation of a New Aerosol Chemical Speciation Monitor (ACSM) System at an Urban Site in Atlanta, GA: The Use of Capture Vaporizer and PM2.5 Inlet. *ACS Earth and Space Chemistry* **2021,** *5*, (10), 2565-2576.
4. Xu, L.; Suresh, S.; Guo, H.; Weber, R. J.; Ng, N. L., Aerosol characterization over the southeastern United States using high-resolution aerosol mass spectrometry: spatial and seasonal variation of aerosol composition and sources with a focus on organic nitrates. *Atmospheric Chemistry and Physics* **2015,** *15*, (13), 7307-7336.
5. DeCarlo, P. F.; Kimmel, J. R.; Trimborn, A.; Northway, M. J.; Jayne, J. T.; Aiken, A. C.; Gonin, M.; Fuhrer, K.; Horvath, T.; Docherty, K. S., Field-deployable, high-resolution, time-of-flight aerosol mass spectrometer. *Analytical chemistry* **2006,** *78*, (24), 8281-8289.
6. Canagaratna, M.; Jayne, J.; Jimenez, J.; Allan, J.; Alfarra, M.; Zhang, Q.; Onasch, T.; Drewnick, F.; Coe, H.; Middlebrook, A., Chemical and microphysical characterization of ambient aerosols with the aerodyne aerosol mass spectrometer. *Mass spectrometry reviews* **2007,** *26*, (2), 185-222.
7. Zhang, Q.; Jimenez, J. L.; Canagaratna, M. R.; Ulbrich, I. M.; Ng, N. L.; Worsnop, D. R.; Sun, Y., Understanding atmospheric organic aerosols via factor analysis of aerosol mass spectrometry: a review. *Analytical and bioanalytical chemistry* **2011,** *401*, (10), 3045-3067.
8. Ulbrich, I.; Canagaratna, M.; Zhang, Q.; Worsnop, D.; Jimenez, J., Interpretation of organic components from Positive Matrix Factorization of aerosol mass spectrometric data. *Atmospheric Chemistry and Physics* **2009,** *9*, (9), 2891-2918.
9. Cubison, M.; Ortega, A.; Hayes, P.; Farmer, D.; Day, D.; Lechner, M.; Brune, W.; Apel, E.; Diskin, G.; Fisher, J., Effects of aging on organic aerosol from open biomass burning smoke in aircraft and laboratory studies. *Atmospheric Chemistry and Physics* **2011,** *11*, (23), 12049-12064.
10. Jimenez, J. L.; Canagaratna, M.; Donahue, N.; Prevot, A.; Zhang, Q.; Kroll, J. H.; DeCarlo, P. F.; Allan, J. D.; Coe, H.; Ng, N., Evolution of organic aerosols in the atmosphere. *Science* **2009,** *326*, (5959), 1525-1529.
11. Janssen, N. A. H.; Hoek, G.; Simic-Lawson, M.; Fischer, P.; Bree, L. v.; Brink, H. t.; Keuken, M.; Atkinson, R. W.; Anderson, H. R.; Brunekreef, B.; Cassee, F. R., Black Carbon as an Additional Indicator of the Adverse Health Effects of Airborne Particles Compared with PM10 and PM2.5. *Environmental Health Perspectives* **2011,** *119*, (12), 1691-1699.
12. Lin, W.; Huang, W.; Zhu, T.; Hu, M.; Brunekreef, B.; Zhang, Y.; Liu, X.; Cheng, H.; Gehring, U.; Li, C.; Tang, X., Acute Respiratory Inflammation in Children and Black Carbon in Ambient Air before and during the 2008 Beijing Olympics. *Environmental Health Perspectives* **2011,** *119*, (10), 1507-1512.

13. Shan, X.; Liu, L.; Li, G.; Xu, K.; Liu, B.; Jiang, W., PM2.5 and the typical components cause organelle damage, apoptosis and necrosis: Role of reactive oxygen species. *Science of The Total Environment* **2021,** *782*, 146785.
14. Zhu, J.; Shang, J.; Chen, Y.; Kuang, Y.; Zhu, T., Reactive oxygen species-related inside-to-outside oxidation of soot particles triggered by visible-light irradiation: physicochemical property changes and oxidative potential enhancement. *Environmental Science & Technology* **2020,** *54*, (14), 8558-8567.
15. Zhu, J.; Shang, J.; Zhu, T., A new understanding of the microstructure of soot particles: The reduced graphene oxide-like skeleton and its visible-light driven formation of reactive oxygen species. *Environmental Pollution* **2021,** *270*, 116079.
16. Chen, Y.; Takeuchi, M.; Nah, T.; Xu, L.; Canagaratna, M. R.; Stark, H.; Baumann, K.; Canonaco, F.; Prévôt, A. S.; Huey, L. G., Chemical characterization of secondary organic aerosol at a rural site in the southeastern US: insights from simultaneous high-resolution time-of-flight aerosol mass spectrometer (HR-ToF-AMS) and FIGAERO chemical ionization mass spectrometer (CIMS) measurements. *Atmospheric Chemistry and Physics* **2020,** *20*, (14), 8421-8440.
17. Lee, B. H.; Lopez-Hilfiker, F. D.; Mohr, C.; Kurtén, T.; Worsnop, D. R.; Thornton, J. A., An iodide-adduct high-resolution time-of-flight chemical-ionization mass spectrometer: Application to atmospheric inorganic and organic compounds. *Environmental science & technology* **2014,** *48*, (11), 6309-6317.
18. Lopez-Hilfiker, F.; Mohr, C.; Ehn, M.; Rubach, F.; Kleist, E.; Wildt, J.; Mentel, T. F.; Lutz, A.; Hallquist, M.; Worsnop, D., A novel method for online analysis of gas and particle composition: description and evaluation of a Filter Inlet for Gases and AEROsols (FIGAERO). *Atmospheric Measurement Techniques* **2014,** *7*, (4), 983-1001.
19. Stark, H.; Yatavelli, R. L.; Thompson, S. L.; Kimmel, J. R.; Cubison, M. J.; Chhabra, P. S.; Canagaratna, M. R.; Jayne, J. T.; Worsnop, D. R.; Jimenez, J. L., Methods to extract molecular and bulk chemical information from series of complex mass spectra with limited mass resolution. *International Journal of Mass Spectrometry* **2015,** *389*, 26-38.
20. Dührkop, K.; Shen, H.; Meusel, M.; Rousu, J.; Böcker, S., Searching molecular structure databases with tandem mass spectra using CSI: FingerID. *Proceedings of the National Academy of Sciences* **2015,** *112*, (41), 12580-12585.
21. Dührkop, K.; Fleischauer, M.; Ludwig, M.; Aksenov, A. A.; Melnik, A. V.; Meusel, M.; Dorrestein, P. C.; Rousu, J.; Böcker, S., SIRIUS 4: a rapid tool for turning tandem mass spectra into metabolite structure information. *Nature methods* **2019,** *16*, (4), 299-302.
22. Ruggeri, G.; Takahama, S., Development of chemoinformatic tools to enumerate functional groups in molecules for organic aerosol characterization. *Atmospheric Chemistry and Physics* **2016,** *16*, (7), 4401-4422.
23. Ditto, J. C.; Joo, T.; Slade, J. H.; Shepson, P. B.; Ng, N. L.; Gentner, D. R., Nontargeted Tandem Mass Spectrometry Analysis Reveals Diversity and Variability in Aerosol Functional Groups across Multiple Sites, Seasons, and Times of Day. *Environmental Science & Technology Letters* **2020,** *7*, (2), 60-69.
24. Debus, B.; Takahama, S.; Weakley, A. T.; Seibert, K.; Dillner, A. M., Long-Term Strategy for Assessing Carbonaceous Particulate Matter Concentrations from Multiple

Fourier Transform Infrared (FT-IR) Instruments: Influence of Spectral Dissimilarities on Multivariate Calibration Performance. *Applied Spectroscopy* **2019,** *73*, (3), 271-283.
25. Takahama, S.; Johnson, A.; Russell, L. M., Quantification of carboxylic and carbonyl functional groups in organic aerosol infrared absorbance spectra. *Aerosol Science and Technology* **2013,** *47*, (3), 310-325.
26. Boris, A. J.; Takahama, S.; Weakley, A. T.; Debus, B. M.; Fredrickson, C. D.; Esparza-Sanchez, M.; Burki, C.; Reggente, M.; Shaw, S. L.; Edgerton, E. S., Quantifying organic matter and functional groups in particulate matter filter samples from the southeastern United States–Part 1: Methods. *Atmospheric Measurement Techniques* **2019,** *12*, (10), 5391-5415.
27. Kroll, J. H.; Donahue, N. M.; Jimenez, J. L.; Kessler, S. H.; Canagaratna, M. R.; Wilson, K. R.; Altieri, K. E.; Mazzoleni, L. R.; Wozniak, A. S.; Bluhm, H., Carbon oxidation state as a metric for describing the chemistry of atmospheric organic aerosol. *Nature chemistry* **2011,** *3*, (2), 133-139.
28. George, K. M.; Ruthenburg, T. C.; Smith, J.; Yu, L.; Zhang, Q.; Anastasio, C.; Dillner, A. M., FT-IR quantification of the carbonyl functional group in aqueous-phase secondary organic aerosol from phenols. *Atmospheric Environment* **2015,** *100*, 230-237.
29. Krapf, M.; El Haddad, I.; Bruns, E. A.; Molteni, U.; Daellenbach, K. R.; Prévôt, A. S.; Baltensperger, U.; Dommen, J., Labile peroxides in secondary organic aerosol. *Chem* **2016,** *1*, (4), 603-616.
30. Yazdani, A.; Dudani, N.; Takahama, S.; Bertrand, A.; Prévôt, A. S.; El Haddad, I.; Dillner, A. M., Characterization of primary and aged wood burning and coal combustion organic aerosols in an environmental chamber and its implications for atmospheric aerosols. *Atmospheric Chemistry and Physics* **2021,** *21*, (13), 10273-10293.
31. Tuet, W. Y.; Chen, Y.; Fok, S.; Gao, D.; Weber, R. J.; Champion, J. A.; Ng, N. L., Chemical and cellular oxidant production induced by naphthalene secondary organic aerosol (SOA): effect of redox-active metals and photochemical aging. *Scientific reports* **2017,** *7*, (1), 1-10.
